# Neuronal guidance factor Sema3A inhibits neurite ingrowth and prevents chondrocyte hypertrophy in the degeneration of knee cartilage in mice, monkeys and humans

**DOI:** 10.1038/s41413-024-00382-0

**Published:** 2025-01-02

**Authors:** Shishu Huang, Dashuang Gao, Zhenxia Li, Hongchen He, Xi Yu, Xuanhe You, Diwei Wu, Ze Du, Jiancheng Zeng, Xiaojun Shi, Qinshen Hu, Yong Nie, Zhong Zhang, Zeyu Luo, Duan Wang, Zhihe Zhao, Lingli Li, Guanglin Wang, Liping Wang, Zongke Zhou, Di Chen, Fan Yang

**Affiliations:** 1 Department of Orthopedics Surgery and Orthopedic Research Institute, National Clinical Research Center for Geriatrics, West China Hospital, Sichuan University, Chengdu, China; 2 The Guangdong Provincial Key Laboratory of Brain Connectome and Behavior, CAS Key Laboratory of Brain Connectome and Manipulation, the Brain Cognition and Brain Disease Institute (BCBDI), Shenzhen Institutes of Advanced Technology, Chinese Academy of Sciences; Shenzhen-Hong Kong Institute of Brain Science-Shenzhen Fundamental Research Institutions, Shenzhen, China; 3 University of Chinese Academy of Sciences, Beijing, China; 4 The seventh Clinical Medical School of Guangzhou University of Chinese Medicine, Bao’an District TCM Hospital, Shenzhen, Guangdong China; 5 Department of Orthodontics, Shanghai Ninth People’s Hospital, Shanghai Jiao Tong University School of Medicine; College of Stomatology, Shanghai Jiao Tong University; National Center for Stomatology; National Clinical Research Center for Oral Diseases, Shanghai, China; 6 Rehabilitation Medicine Center, West China Hospital, Sichuan University, Chengdu, China; 7 State Key Laboratory of Oral Diseases, National Clinical Research Center for Oral Diseases, West China Hospital of Stomatology, Sichuan University, Chengdu, China; 8 Department of Nursing, West China Hospital, Sichuan University, Chengdu, China; 9 Research Center for Computer-aided Drug Discovery, Shenzhen Institute of Advanced Technology, Chinese Academy of Sciences, Shenzhen, China

**Keywords:** Bone, Neurophysiology

## Abstract

Osteoarthritis (OA) is a degenerative joint disease accompanied with the loss of cartilage and consequent nociceptive symptoms. Normal articular cartilage maintains at aneural state. Neuron guidance factor Semaphorin 3A (Sema3A) is a membrane-associated secreted protein with chemorepulsive properties for axons. However, the role of Sema3A in articular cartilage is still not clear. In the present studies, we investigated the functions of Sema3A in OA development in mice, non-human primates, and patients with OA. Sema3A has a protective effect on cartilage degradation, validated by the organoid culture in vitro and confirmed in chondrocyte-specific Sema3A conditional knockout mice. We demonstrated that Sema3A is a key molecule in maintaining cartilage homeostasis from chondrocyte hypertrophy via activating the PI3K pathway. The potential usage of Sema3A for OA treatment was validated in mouse and Rhesus macaque OA models through intra-articular injection of Sema3A, and also in patients by administering Sema3A containing platelet-rich plasma into the knee joints. Our studies demonstrated that Sema3A exerts a critical role in inhibiting neurite ingrowth and preventing chondrocyte hypertrophy in cartilage, and could be potentially used for OA treatment.

## Introduction

The evolutionary transition in humans from walking on all fours to walking upright contributed to our ability to traverse multiple terrain types, which arguably led to human civilization.^1–3^ During our evolution, the knee joint has played an important role and is extremely important in modern life. However, intensive overuse and aging of knee joints in modern life are generally accompanied with osteoarthritis (OA), a progressive degeneration of knee joints, which is commonly associated with a range of pathological changes, including chondrocyte hypertrophy.^4,5^ This has become one of the most painful diseases reported worldwide.^6,7^ Interestingly, OA patients manifest different clinical symptoms in the OA degeneration process. Nociceptive symptoms are obvious in the first stage, as is limited action of the knee joint in the second stage. The pathological mechanisms of the two manifestations are unknown. In particular, how nociceptive symptoms arise in the progressive degeneration of joint cartilage is not clear.

Normal joint cartilage is aneural tissue and functions anatomically as a lubricant. Interestingly, neuronal ingrowths have been observed in cartilage degeneration and the consequent pain felt is thought to be associated with peripheral and central sensitization.^8^ Peripheral sensitization occurs in all structures of the joint except in normal cartilage, including ligaments, the fibrous capsule, osteochondral junctions, and meniscuses. During central sensitization, superficial and deep dorsal horn tissue in several segments along the spinal cord are found to project from large joints to the brain.^8–10^ Nerve growth factor (NGF) is involved in the generation of OA pain, which significantly increases the knee-extension-evoked firing of spinal neurons.^11–17^ Conversely, neutralizing or blocking NGF activity exerts an analgesic effect accordingly, which is associated with a reduction in the number of subchondral osteoclasts in subchondral bone remodeling.^12,14^ It has been shown that subchondral bone releases axonal guidance molecules and induces axonal growth and sensory innervation, which mediate OA pain.^18^ However, to date, it is not clear whether the chondrocytes within the noncalcified articular cartilage are responsive to nerve factors. The mechanism underlying cartilage degeneration and induced nociceptive symptoms is also unknown.

In this study, we compared quadrupedal, semi-erect, and erect knees, and identified one neuron guidance factor, Semaphorin 3A (Sema3A), a membrane-associated secreted protein with chemorepulsive properties relating to axons.^19,20^ In the *Sox9-CreER*^*T2*^*: Semaphorin 3a*^*flox/flox*^ mouse model (Sema3A^KO^), expression of chondrocytic Sema3A was low early in the OA process. This increased neuronal infiltration to the cartilage, which inhibited neural collapse and further elevated nociceptive symptoms. Additionally, Sema3A maintained cartilage homeostasis from chondrocyte hypertrophy via the PI3K pathway, which has recently been reported in the synovial hyperplasia in OA.^21^ Furthermore, Sema3A consistently showed potential in the arrest of OA progression in anterior cruciate ligament transection (ACLT) mice, and senile OA *Rhesus* macaques.^22^ In humans, treatment with the platelet-rich plasma (PRP) containing sema3A results in a significant reduction of OA-induced nociceptive symptoms. Our study thus shows that neuronal guidance factor Sema3A exerted critical roles in inhibiting neurite ingrowth and preventing chondrocytic hypertrophy in the degeneration process of knee joints.

## Results

### Elevated neural ingrowth and Semaphorin 3A expression in OA knee cartilage

**Fig. 1:**
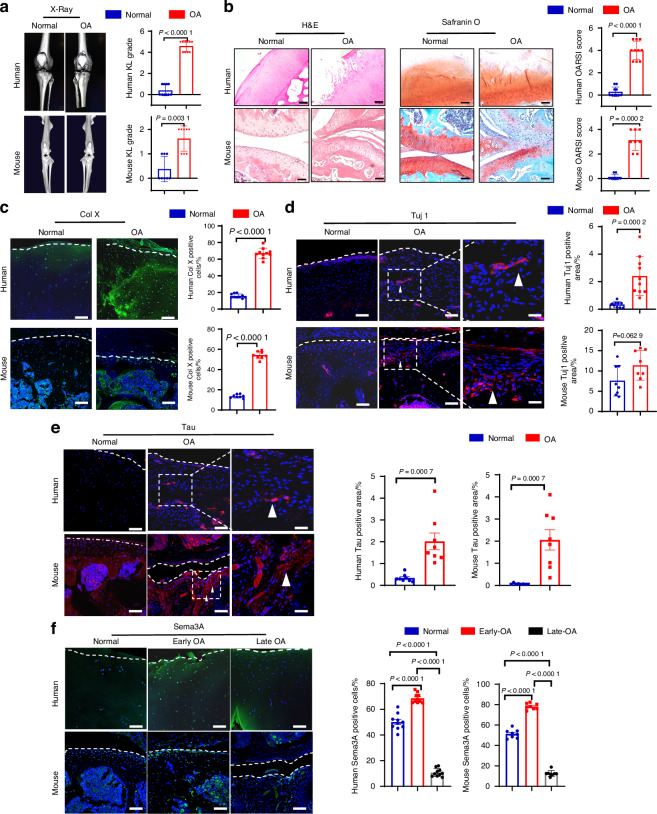
Elevated expression of neural factors of human and mice knee cartilage during OA progression. **a** *Left*, orthotopic view of 3D reconstruction CT of normal and OA knee joints in humans and mice. *Right*, Kellgren–Lawrence (KL) analysis of the joint space between normal control (*n* = 10 patients, *n* = 8 mice) and OA (*n* = 10 patients, *n* = 8 mice) groups. A two-tailed Mann–Whitney U test was used for statistical analysis. Mean with SD. A representative result from five independent experiments is shown. **b** *Left*, H&E staining of cartilage in normal control and OA groups. Scale bar, 200 µm (human), 200 µm (mouse). Middle, Safranin O staining of the cartilage in normal control and OA groups. Scale bar, 200 µm(human), 100 µm(mouse). *Right*, OARSI scores from normal control (*n* = 10 individuals, *n* = 8 mice) and OA (*n* = 10 individuals, *n* = 8 mice) groups. A two-tailed Mann–Whitney U test was used for statistical analysis. Mean with SD. A representative result from five independent experiments is shown. **c** Immunofluorescence staining of type X collagen (Col X) in the normal control (*n* = 10 individuals, *n* = 8 mice) and OA (*n* = 10 individuals, *n* = 8 mice) groups. Quantitative analysis of the cells of positive Col X cells per cartilage was performed among groups. A two-tailed unpaired student’s t-test was used for statistical analysis. Mean with SD. Scale bar, 50 µm. **d** Immunofluorescence staining of Tuj1 in the normal control (*n* = 10 individuals, *n* = 8 mice) and OA (*n* = 10 individuals, *n* = 8 mice) groups. Scale bar, 50 µm. Quantitative analysis of the area of positive Tuj1 per cartilage area (mm^2^) was performed among groups. High magnification of Tuj1 staining in knee cartilage was shown in respective groups (arrowhead). A two-tailed unpaired student’s t-test was used for statistical analysis. Mean with SD. Scale bar, 25 µm. **e** Immunofluorescence staining of Tau in the normal control (*n* = 8 individuals, *n* = 8 mice) and OA (*n* = 8 individuals, *n* = 8 mice) groups. Scale bar, 50 µm. Quantitative analysis of the area of positive Tau per cartilage area (mm^2^) was performed among groups. High magnification of Tau staining in knee cartilage was shown in respective groups (arrowhead). A two-tailed unpaired student’s t-test was used for statistical analysis. Mean with SD. Scale bar, 25 µm. **f** Immunofluorescence staining of Semaphorin 3A (Sema3A) in the normal control (*n* = 10 individuals, *n* = 8 mice), early OA (*n* = 10 individuals, *n* = 8 4-weeks OA mice), late OA (*n* = 10 individuals, *n* = 8 12-weeks OA mice) groups. Quantitative analysis of the number of positive Sema3a cells in cartilage was performed among groups. Scale bar, 50 µm. A two-tailed unpaired student’s t-test was used for statistical analysis. Mean with SD. A representative result from five independent experiments is shown. OA osteoarthritis. OARSI score Osteoarthritis Research Society International scoring system

To investigate the association between OA and neural ingrowth, we utilized knee cartilage from both OA patients (Table 1) and mice that had received anterior cruciate ligament transection (ACLT) surgery. Three-dimensional (3D) micro-computed tomography (μCT) analysis showed that knees from OA patients and ACLT mice had narrowing of joint space and more marginal osteophytes compared to normal individuals and normal wild-type mice, respectively, reflected by a 50% higher Kellgren–Lawrence (KL) score in the OA groups compared to the normal groups (Fig. 1a). Images from patients and ACLT mice (OA) show corruption in the cartilage surface, disruption of connectivity and microarchitectural structure in the cartilage layer, disorganized chondrocyte morphology, and substantially higher levels of broken tidemark compared to normal humans or mice (Fig. 1b). We used Hematoxylin and Eosin (*H&E*), which stained cell nuclei blue-purple and cartilage matrix pink, and we used Safranin O to stain proteoglycans orange/red (Fig. 1b). We evaluated the severity of degeneration using OARSI analysis, and scores were more than 30% higher in the cartilage of patients and ACLT mice compared to normal humans or mice, respectively (Fig. 1b). Type X collagen (Col X) staining revealed that the number of hypertrophic chondrocytes was higher in cartilage tissue that had degenerated, and immunofluorescence (IF) revealed that the number of Col X positive cells was significantly higher in the cartilage of patients and ACLT mice compared to normal humans or mice, respectively (Fig. 1c). We also explored neural ingrowth in response to OA using neuron-specific class III beta-tubulin (Tuj1), which stains dendrites with axons^23^ and using a Tau stain, which only stains axons.^24^ The percentage of positive Tuj1 and Tau stained areas were both significantly higher in the cartilage of patients with OA and ACLT mice received the ACLT surgery compared to normal humans and mice (Fig. 1d, e). To further observe the genetic expression profile of chondrocytes in OA, RNA sequencing was performed and revealed massive changes in transcriptomes, which included an up-regulation of Sema3A (Fig. S1A, B). Gene ontology (GO) analysis suggested that the molecular pathway of muscular system processes and muscle contraction in chondrocytes are amplified in OA, which is further associated with cartilage degeneration (Fig. S2). In the early stage of OA, there is a temporary increase of Sema3a expression, however, the expression of the Seme3A decreases continuously in the OA course due to the loss of chondrocytes (Fig. 1f). We also show the Sema3A receptor of Neuropilin 1(NPR1) and Plexin-A expression in the human and mouse articular cartilage by immunostaining. NPR1, Plexin-A, and Sema3A are co-expressed in mouse and human articular chondrocytes (Fig. S3A–D). We performed immunostaining to investigate the abnormal phosphorylation of Tau protein in osteoarthritis (OA), where a typical phosphorylation site found was serine residue 231 (referred to as Tau-P231), which showed significant expression (Fig. S3E). Additionally, through FISH staining, we confirmed that Sema3a is expressed in chondrocytes located in OA cartilage (Fig. S3F). We investigated the expression of Sema3A in various tissues of the joints and found that Sema3A is expressed in cartilage, synovium, subchondral bone, and ligament (Fig. S4A, B). Based on the significant changes of Sema3A in the development of OA, we hypothesized that Sema3A may have both roles of chondroprotection and preventing nerve infiltration into cartilage.

**Table 1:** Summary of OA patients from whom cartilage samples were collected for analysis

Patient No. OA group	Age	Gender	KL	Patient No. Control group	Age	Gender	KL
1	66	M	5	1	38	M	1
2	71	M	5	2	55	M	2
3	67	M	4	3	49	M	1
4	74	M	5	4	50	M	2
5	70	M	4	5	47	M	1
6	74	M	4	6	51	M	1
7	65	M	4	7	55	M	2
8	72	M	5	8	53	M	2
9	71	M	4	9	47	M	1
10	73	M	5	10	52	M	2

*KL* Kellgren–Lawrence grading scale

### Deletion of Sema3A in chondrocytes promotes nerve ingrowth and affects OA progression

**Fig. 2:**
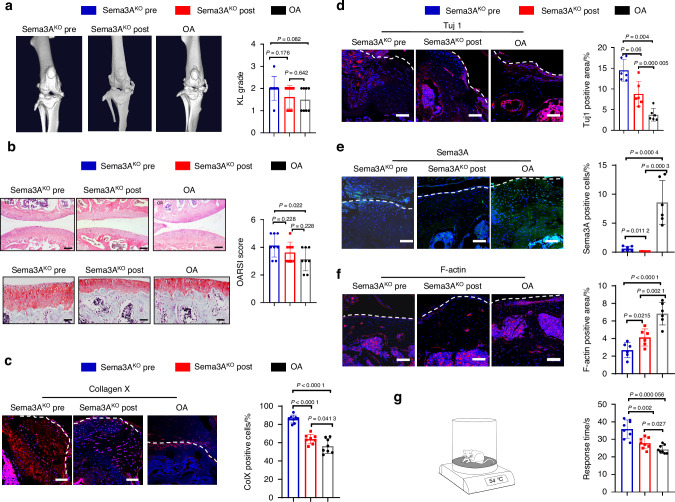
Conditional knockout of Sema3A in chondrocytes before and after ACLT surgery display different knee phenotypes. **a** *Left*, orthotopic view of 3D reconstruction CT images of the Sema3A knockout before ACLT surgery (Sema3A^KO^ Pre), Sema3A knockout after ACLT surgery (Sema3A^KO^ Post) and control ACLT groups. *Right*, Kellgren–Lawrence analysis of the joint space in Sema3A^KO^ Pre (*n* = 8), Sema3A^KO^ Post (*n* = 8), and ACLT (*n* = 8) groups. A two-tailed unpaired student’s t-test was used for statistical analysis. Mean with SD. A representative X-ray image from five independent experiments is shown. **b** *Top*, H&E staining of the cartilage in Sema3A^KO^ Pre, Sema3A^KO^ Post, and ACLT groups. Scale bar, 100 µm. *Bottom*, Safranin O staining of cartilage in Sema3A^KO^ Pre, Sema3A^KO^ Post, and ACLT groups. Scale bar, 50 µm. *Right*, The OARSI score of the Sema3A^KO^ Pre (*n* = 8), Sema3A^KO^ Post (*n* = 8), and ACLT (*n* = 8) groups. Safranin O staining of proteoglycan (red) and bone (blue) in sagittal sections of the distal femur compartment. A two-tailed unpaired student’s t-test was used for statistical analysis. Mean with SD. A representative image of each group from five independent experiments is shown. **c** Immunofluorescence staining of Collagen X in Sema3A^KO^ Pre (*n* = 8), Sema3A^KO^ Post (*n* = 8), ACLT (*n* = 8) groups and the quantitative analysis of the cells of Collagen X positive cells per cartilage. Scale bar, 50 µm. A two-tailed unpaired student’s t-test was used for statistical analysis. Mean with SD. **d** Immunofluorescence staining of Tuj1 in Sema3A^KO^ Pre (*n* = 6), Sema3A^KO^ Post (*n* = 6), ACLT (*n* = 6) groups and the quantitative analysis of the area of Tuj1 positive area per cartilage area (mm^2^). Scale bar, 50 µm. A two-tailed unpaired student’s t-test was used for statistical analysis. Mean with SD. A representative result from five independent experiments is shown. **e** Immunofluorescence staining of Sema3A in Sema3A^KO^ Pre (*n* = 6), Sema3A^KO^ Post (*n* = 6), ACLT (*n* = 6) groups and the quantitative analysis of the number of semaphorin-positive cells per cartilage. Scale bar, 50 µm. A two-tailed unpaired student’s t-test was used for statistical analysis. Mean with SD. A representative result from five independent experiments is shown. **f** Immunofluorescence staining of F-actin in Sema3A^KO^ Pre (*n* = 6), Sema3A^KO^ Post (*n* = 6), ACLT (*n* = 6) groups and the quantitative analysis of the area of F-actin^+^ per cartilage area (mm^2^). Scale bar, 50 µm. A two-tailed unpaired student’s t-test was used for statistical analysis. Mean with SD. A representative result from five independent experiments is shown. **g** Quantitative analysis of the latency to lick a hind paw following mice from different groups (Sema3A^KO^ Pre (*n* = 8), Sema3A^KO^ Post (*n* = 8), ACLT (*n* = 8)) being placed on a 54 °C plate. A two-tailed unpaired student’s t-test was used for statistical analysis. Mean with SD. ACLT, anterior cruciate ligament transection. OARSI score, Osteoarthritis Research Society International scoring system

We next tested whether neuronal guidance factor Sema3A is involved in OA progression and whether it is associated with OA pain. We crossbred *SRY*-Box Transcription Factor 9 (Sox9)-CreER^T2^ knock-in mice with Sema3A^flox/flox^ mice to generate conditional Sema3A^KO^ mice (Fig. S5A–D). We successfully knocked out Sema3A in the cartilage layer (Fig. 2e, Fig. S5F). The deficiency of Sema3A in chondrocytes was induced by knockout of Sema3A in chondrocytes before ACLT surgery (Sema3A^KO^ Pre) resulting in more pronounced joint space narrowing and more osteophytes compared to Cre-negative mice that received ACLT surgery (OA), reflected by a 20% higher KL score in the Sema3A^KO^ Pre group compared to the OA group (Fig. 2a). Conversely, the difference between the KL score in the Sema3A knockout in chondrocytes post-ACLT (Sema3A^KO^ Post) group was not significantly different from the ACLT group’s KL score (Fig. 2a). Additionally, Sema3A^KO^ Pre mice had more severe corruption of cartilage surface, more disruption of connectivity and microarchitectural structure in the cartilage layer, higher levels of chondrocyte-morphology disorganization, and substantially higher levels of broken tidemark, relative to the OA group, as revealed by *H&E* and Safranin O staining and analyzed by OARSI scoring (Fig. 2b). There was no significant difference in OARSI scoring between Sema3A^KO^ Post mice and ACLT mice (Fig. 2b). Compared to OA and Sema3A^KO^ Post group, Sema3A^KO^ Pre mice tended to have more positive Col X cells (Fig. 2c). This suggests that Sema3A expression levels are associated with Col X expression levels. Additionally, Sema3A^KO^ Pre mice and Sema3A^KO^ Post mice had more positive Tuj1 signals than mice in the OA group (Fig. 2d), indicating increased nerve ingrowth following sema3A knockout. We also measured the expression of F-actin, a marker of neural collapse, and found that both Sema3A^KO^ Pre and Sema3A^KO^ Post mice had less F-actin than ACLT mice (Fig. 2f). We then measured knee function, in particular, induced nociceptive symptoms, by testing latency to lick a hind paw following contact with a 54 °C hotplate. The Sema3A^KO^ Pre group had significantly longer latency than both the Sema3A^KO^ Post group and OA group, indicating that nociceptive symptoms were more severe in the Sema3A^KO^ Pre mice (Fig. 2g). Taken together, our data demonstrate that knockout of Sema3A at different stages of OA leads to distinguishable knee joint phenotypes and that Sema3A^KO^ Pre mice had a more severe OA progression compared to Sema3A^KO^ Post mice and ACLT mice.

### Sema3A expression in chondrocytes regulates both nerve ingrowth and cartilage hypertrophy

**Fig. 3:**
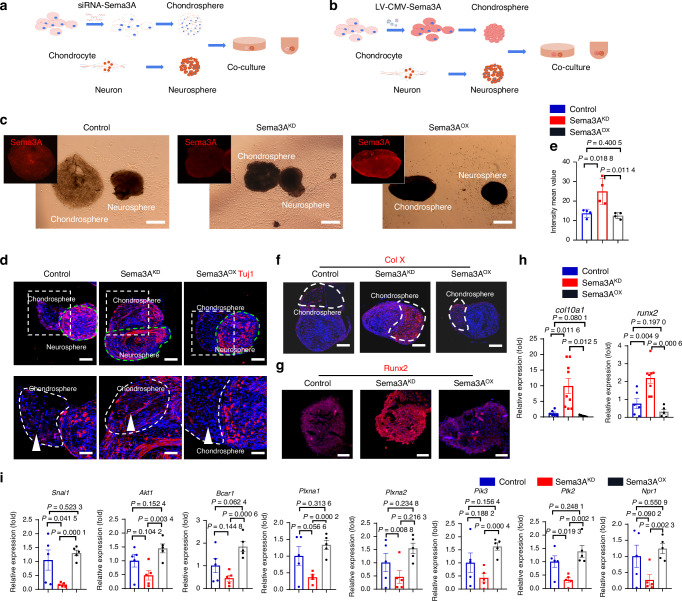
Bidirectional regulation of Sema3A expression in chondrocytes regulates both nerve ingrowth and chondrocyte hypertrophy. **a** Schematic program of the generation of chondrosphere and dorsal root ganglion (DRG) neurosphere. Sema3A was knocked down in the chondrocytes by siRNA-Sema3A. **b** Schematic program of the generation of chondrosphere and DRG neurosphere. Sema3A was overexpressed in the chondrosphere by lentivirus CMV-Sema3A-GFP. **c** The co-culture of chondrospheres and neurospheres, and morphological changes of neuritis growth in the normal control group (*left*) with normal Sema3A expression in the chondrosphere, Sema3A^KD^ group (*middle*) with decreased Sema3A expression in chondrosphere; Sema3A^OX^ group (*right*) with overexpression of sema3A in chondrosphere. Scale bar, 500 µm. **d** Immunofluorescence staining of Tuji1 in the normal control group with control chondrosphere (*left*), Sema3A^KD^ group with Sema3A knockdown in chondrosphere (*middle*), sema3A^OX^ group with Sema3A overexpressed in chondrosphere (*right*). Scale bar, 100 µm. High magnification of Tuji1 staining in chondrosphere was shown in respective groups (bottom panel, arrowhead). Scale bar, 50 µm. **e** Quantitative analysis of the intensity of Tuj1 per chondrosphere area (mm^2^). A two-tailed student’s t-test test was used for statistical analysis. Mean with SD. **f** Immunofluorescence staining of type X collagen (Col X) in control chondrocyte spheres (*left*), Sema3A knockdown chondrocyte spheres (*middle*) and Sema3A overexpressed chondrocyte spheres (*right*). Scale bar, 50 µm. **g** Immunofluorescence staining of Runx2 in control chondrosphere (*left*), Sema3a knockdown chondrosphere (*middle*), and Sema3a overexpressed chondrosphere (*right*). Scale bar, 50 µm. **h** Expression of *Col10a1* and *Runx2* genes in normal control (*n* = 6), sema3A^KD^ (*n* = 9), and sema3A^OX^ (*n* = 5) groups, A two-tailed student’s t-test was used for statistical analysis. Mean with SD. **i** Expression of genes involved in the Semaphorin 3A pathway was tested by quantitative RT–PCR (*n* = 5 for Ctrl, *n* = 5 for the knockdown, *n* = 5 for overexpression). A two-tailed student’s t-test was used for statistical analyses. All data are reported as the mean ± SD

To explore the molecular mechanism of Sema3A in the progression of cartilage degeneration, we established a spheroid co-culture system in vitro (Fig. 3a, b). Human knee chondrocytes and rat dorsal root ganglion (DRG) neurons were cultured separately and then shaken to form 3D spheres. Knockdown of Sema3A in chondrocytes was achieved with siRNA-Sema3A (Fig. 3a), and overexpression of Sema3A was achieved with LV-CMV-Sema3A-Ef1a-EGFP (Fig. 3b). Normal, down-regulated, and up-regulated Sema3a expression levels were successfully obtained, respectively, from normal control, Sema3A^KD^ and Sema3A^OX^ groups (Fig. 3c). We then used Tuj1 staining to evaluate nerve ingrowth between groups and found that low-expression of chondrocyte Sema3A had the most nerve-terminal growth into chondrospheres and overexpression of chondrospheres Sema3A had the least (Fig. 3d). Quantitative analysis confirmed a higher number of nerve terminals in the Sema3A^KD^ group compared to the Sema3A^OX^ and control groups (Fig. 3e), suggesting that a low level of Sema3A in the chondrosphere enhances nerve-terminal growth, whereas higher levels are inhibitory. Importantly, modulation of Sema3A expression also affected the expression of type X collagen and Runx2 in chondrospheres. We found that low Sema3A expression in the Sema3A^KD^ group led to higher chondrocytic expression of Col X and Runx2 than in the control group, whereas overexpression of sema3A in the Sema3A^OX^ group led to lower chondrocyte expression of Col X and Runx2 compared with Sema3A^KD^ group (Fig. 3f–h). We then used quantitative PCR to further dissect gene-expression changes related to semaphorin intracellular signaling (Fig. 3i). We found a consistent down-regulation of genes in the PI3K pathway in the Sema3A^KD^ group, whereas expression levels of these genes were higher in the Sema3a^OX^ group (Fig. 3i). Our quantitative PCR results suggest that Sema3A conjugates to the Sema3A receptor, PlexinA1, stimulated the expression of SNAI1, PI3K, and Runx2, which are associated with expression of type X collagen. The upstream factors *Adgrg3, Esra*, and *Vegfa* have also been quantified by the real-time PCR in control and OA groups. Our results showed that the expression of *Adgrg3* and *Vegfa* as upstream factors increased in the OA group (Fig. S6). In summary, our results suggest that Sema3A is involved in the maintenance of chondrocytes from hypertrophy. In contrast, down-regulation of Sema3A induced both chondrocyte hypertrophy and nerve ingrowth into cartilage.

### Sema3A arrests OA progression in mouse models

**Fig. 4:**
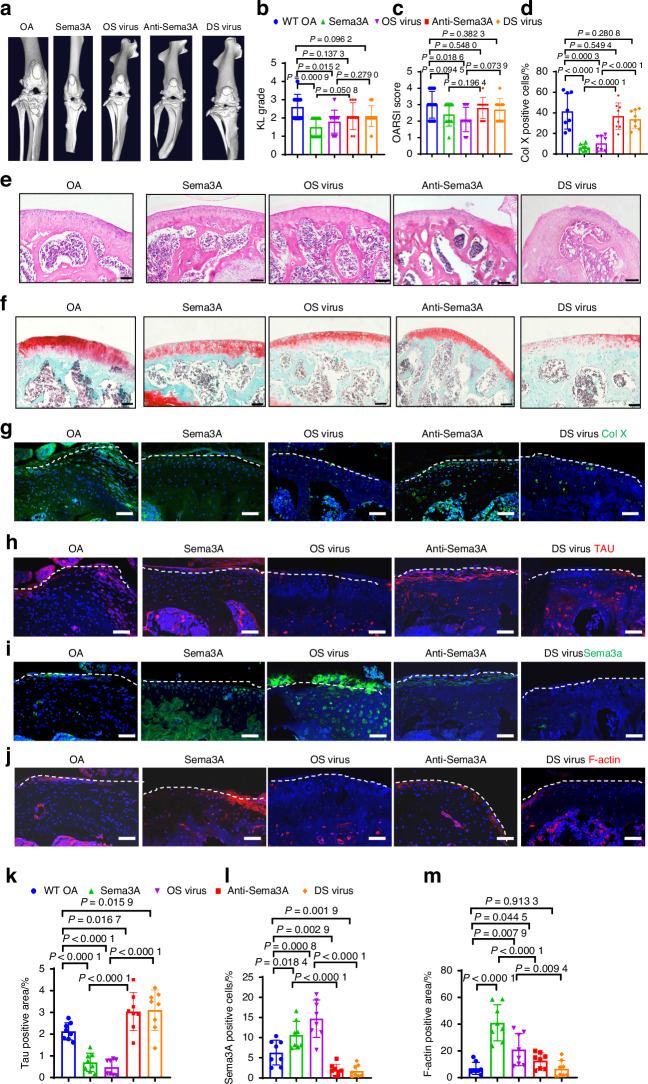
Local administration of Sema3A agents in the articular cartilage arrest degeneration in ACLT mice. **a**, **b** 3D reconstruction of micro-CT and Kellgren–Lawrence analysis of the joint space in OA (*n* = 10), Sema3A protein (*n* = 10), Sema3A overexpressed virus (OS virus) (*n* = 10), Anti-Sema3A antibody (*n* = 10), Sema3A low-expressed virus (DS virus) (*n* = 10) mice groups. Statistical significance was determined by multifactorial ANOVA. All data are reported as the mean ± SD. **c**, **e**, **f** H&E staining, Safranin O staining, and OARSI scores of the OA (*n* = 10), Sema3A protein (*n* = 10), OS virus (*n* = 10), Anti-Sema3A antibody (*n* = 10), DS virus (*n* = 10) mice groups. Statistical significance was determined by multifactorial ANOVA. All data are reported as the mean ± SD. Scale bar, 100 µm. A representative result from five independent experiments is shown. **d**, **g** Immunofluorescence staining of Collagen X in OA (*n* = 8), Anti-Sema3A antibody (*n* = 8), Sema3A protein (*n* = 8), OS virus (*n* = 8), Sema3A DS virus (*n* = 8) mice groups. Statistical significance was determined by multifactorial ANOVA. All data are reported as the mean ± SD. Scale bar, 50 µm. A representative result from five independent experiments is shown. **h**, **k** Immunofluorescence staining of Tau in OA (*n* = 8), Anti-Sema3A antibody (*n* = 8), Sema3A protein (*n* = 8), OS virus (*n* = 8), DS virus (*n* = 8) mice groups. Statistical significance was determined by multifactorial ANOVA. All data are reported as the mean ± SD. Scale bar, 50 µm. A representative result from five independent experiments is shown. **i**, **l** Immunofluorescence staining of Sema3A in OA (*n* = 8), Anti-Sema3A antibody (*n* = 8), Sema3A protein (*n* = 8), OS virus (*n* = 8), DS virus (*n* = 8) mice groups. Statistical significance was determined by multifactorial ANOVA. All data are reported as the mean ± SD. Scale bar, 50 µm. A representative result from five independent experiments is shown. **j**, **m** Immunofluorescence staining of F-actin in OA (*n* = 8), Anti-Sema3A antibody (*n* = 8), Sema3A protein (*n* = 8), OS virus (*n* = 8), DS virus (*n* = 8) mice groups. Statistical significance was determined by multifactorial. ANOVA. All data are reported as the mean ± SD. Scale bar, 50 µm. A representative result from five independent experiments is shown. OARSI score, Osteoarthritis Research Society International scoring system

To further verify the potential of Sema3A in the treatment of OA. We next investigated the therapeutic potential of Sema3A in the treatment of OA using mouse models. First, we identified different groups for comparison and set them up by injecting the following substances into the murine knee cavity: Sema3A protein (Sema3A), overexpressed Sema3A with Lentivirus (OS virus), neutralizing Sema3A antibody (Anti-Sema3A), and down-expressed Sema3A Lentivirus (DS virus). Following micro-CT analysis, we found that, compared to the OA control group, Sema3A protein, and the OS virus groups showed less joint space narrowing, substantially lower marginal osteophytes, and lower KL scores. By contrast, compared to the OA control group, the Anti-Sema3A, and the DS virus groups showed more joint space narrowing, substantially higher marginal osteophytes, and higher KL scores (Fig. 4a, b). The Sema3A and OS virus groups showed less corrupted surface and less disruption of connectivity and microarchitectural structure in the cartilage layer. The Anti-Sema3A and the DS virus groups had a more corrupted surface, more disruption of connectivity, and microarchitectural structure in the cartilage layer. The Sema3A and OS virus groups had substantially fewer broken tidemarks compared with other groups, which was visible following *H&E* and Safranin O staining. This was further confirmed by OARSI scoring (Fig. 4c, e, f). The number of hypertrophic chondrocytes and the number of positive neural markers, revealed by Col X staining and Tau staining, were both significantly lower in the Sema3A and OS virus groups than in the OA groups (Fig. 4d, g, h, k). Additionally, we measured Sema3A expression in chondrocytes of knee joint cartilage and found that the Sema3A group and OS virus group had a significantly higher number of Sema3A positive cells compared to OA group, and the Anti-Sema3A and the DS virus groups had a significantly less number of Sema3A positive cells compared to OA group (Fig. 4i, l). It shows that injection of Sema3A and OS virus can effectively increase the content of Sema3A in cartilage, and Sema3A antibody and DS virus can effectively reduce the expression of Sema3A in cartilage. Which was associated with neural collapse as revealed by F-actin staining, the results showed that Sema3A and OS virus groups F-actin were more expressed (Fig. 4j, m). As shown by the results of Sema3A and F-actin, it is proved that more Sema3A inhibited nerve growth into cartilage. For knee function, measurement of nociceptive symptoms showed that the Sema3A and OS virus groups had higher pain sensitivity, which suggests improved function after Sema3A treatment (Fig. S7B). Sema3A and OS virus groups resulted in increased Sema3A in the articular cartilage area, along with reduced chondrocyte hypertrophy and reduced neural infiltration. These results suggest that Sema3A treatment of OA has a significant chondroprotective effect and maintains the aneural state of cartilage.

### Sema3A enhances knee function in monkey OA models

**Fig. 5:**
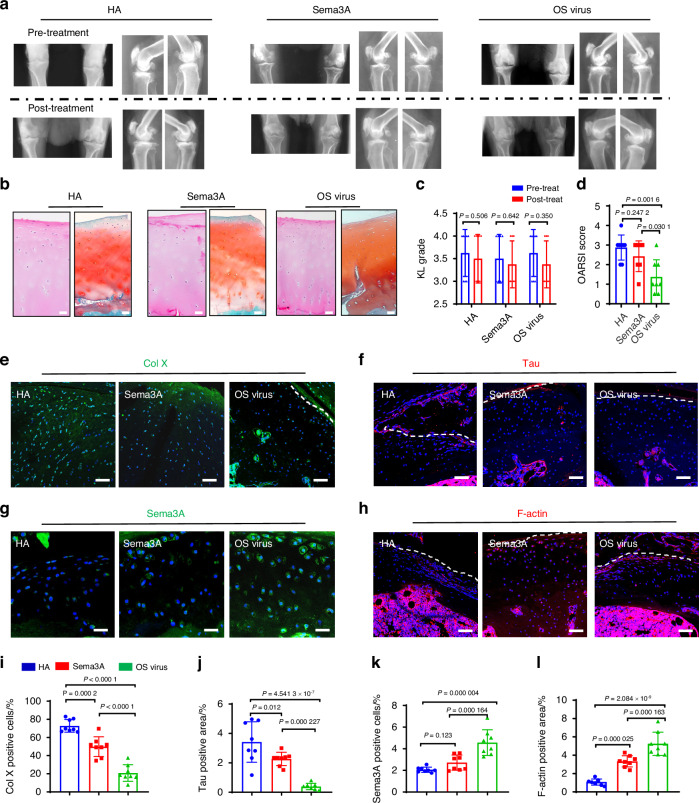
Local administration of Sema3A agents in the articular cartilage arrests the knee joint degeneration in natural OA *Rhesus* macaques. **a**, **c** Representative X-ray images and Kellgren–Lawrence analysis of the joint space in hyaluronate acid (HA) (*n* = 8), Sema3A protein (*n* = 8), Sema3A overexpressed virus (OS Virus) (*n* = 8) Rhesus macaques joint groups. Statistical significance was determined by multifactorial ANOVA. All data are reported as the mean ± SD. **b**, **d** OARSI scores for the HA (*n* = 8), Sema3A protein (*n* = 8), OS Virus (*n* = 8) Rhesus macaques groups. Statistical significance was determined by multifactorial ANOVA. All data are reported as the mean ± SD. A representative result from three independent experiments is shown. Scale bar, 50 µm. **e**–**l** Immunofluorescence staining of Col X (**e**, **i**), Tau (**f**, **j**), Sema3A (**g**, **k**), and F-actin (**h**, **l**) in HA group (*n* = 8), Sema3A protein group (*n* = 8), OS Virus group (*n* = 8) and Rhesus macaques group. All data are reported as the mean ± SD. A representative result from three independent experiments was shown. **e**, **f**, **h** Scale bar, 50 µm. **g** Scale bar, 25 µm. OARSI score, Osteoarthritis Research Society International scoring system

We demonstrated the therapeutic potential of Sema3A in the treatment of OA in mouse models. However, the mouse OA arthritis model is not an ideal arthritis model. In the mouse OA model, the articular cartilage wears too fast and the OA process accelerates. The mouse OA model cannot simulate the slow cartilage wear process in OA patients. *Rhesus* monkeys walk upright like humans. *Rhesus* monkeys also develop arthritis as their articular cartilage wears out with aging, similar to humans. We next implemented a senile OA *Rhesus* macaque model to validate the therapeutic potential of Sema3A in comparison to the clinical agent, HA (Fig. S7A). The Sema3A protein group, overexpressed Sema3A lentivirus group (OS virus), and HA group all had lower KL scores post-treatment compared to pre-treatment 4 weeks after treatment. However, the Sema3A and OS virus groups both had a greater tendency to have lower KL scores compared to the HA group (Fig. 5a, c), suggesting that Sema3A may have a chondroprotective effect on OA treatment. Additionally, we harvested knee-cavity cartilage and analyzed tissue histology, we found that the Sema3A and OS virus groups had lower OARSI scores (Fig. 5b, d). However, the treatment effect of the Sema3A group was not as obvious as that of the OS virus group. Overexpression of Sema3A by the virus can significantly reduce OARSI scores, and the OARSI scores in the OS virus group were half of the HA group. It shows that the effect of re-expressing Sema3A in chondrocytes by the virus is better than injecting Sema3A protein, which may be because there is a large amount of tissue fluid in the joint capsule in OA, which may rapidly degrade the injected Sema3A protein. We also examined the expression of Col X, Tau, and Sema3A in cartilage. The Sema3A and OS virus groups were significantly fewer numbers of Col X cells than the HA group (Fig. 5e, i). The cartilage hypertrophy in the Sema3A and OS virus groups was significantly improved, similar to the results of OARSI scores, and the OS virus group was significantly better than the HA and Sema3A groups. The Sema3A and OS virus groups were significantly fewer numbers of TAU nerve terminals than the HA group (Fig. 5f, j). This shows that Sema3A can significantly inhibit nerve infiltration into cartilage in OA. Similarly, the OS virus group was better than the HA and Sema3A groups. These scores were correlated with the elevated Sema3A expression and positive Sema3A cells (GFP) in the groups (Fig. 5g, k). We found that the expression of Sema3A in the OS virus group was the highest, and it was also the best recovery, indicating that the higher the expression of Sema3A, the better the effect of protecting cartilage. We also associated with neural collapse as revealed by F-actin staining (Fig. 5h, l). Through the results of F-actin, the expression of F-actin in the OS virus group was the highest. Indicating that the more the expression of Sema3A, the more inhibited the infiltration of nerves.

### PRP is rich in Sema3A and enhances human knee function in a randomized controlled trial

The platelet-rich plasma (PRP) has been widely used in the field of osteoarticular diseases and soft tissue injuries. Since semaphorin 3A (Sema3a) was shown to selectively reduce NGF-induced sprouting and neuropathic pain,^17^ we determined the level of Sema3a in PRP and found that PRP plasma is indeed rich in Sema3A: the concentration of Sema3A in PRP is 3–5 times higher than that in normal plasma, (Fig. S8B). To explore the effects of Sema3A on nerve ingrowth, we established a spheroid co-culture system in vitro and found that Sema3A enriched plasma significantly inhibited the ingrowth of the nerve fibers compared with the control serum, however adding 50 ng/mL anti-Sema3a antibody into the PRP significantly blocked the inhibitory effect of PRP serum (Fig. S8C). We treated chondrocytes with soluble SEMA3A and observed that there was no nerve fiber ingrowth into chondrospheres (Fig. S8D). We injected Sema3a (1.2 ng/L) into the joint cavity of OA mice and assessed the pain response using Von Frey testing. The results indicated that the injection of Sema3a alleviated the pain induced by OA in mice (Fig. S8F).

**Fig. 6:**
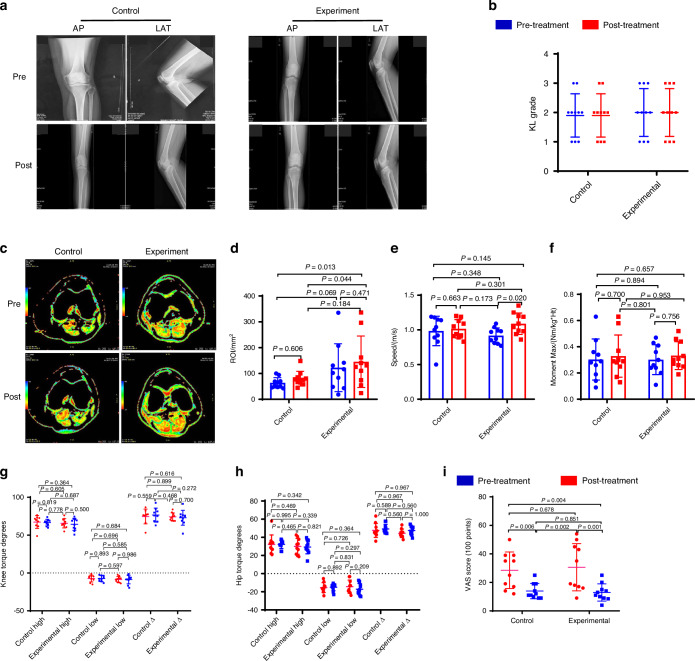
The platelet-rich plasma (PRP) containing Sema3A injection rescues knee cartilage during the normal degenerative process in OA patients. **a** Orthotopic (AP) and lateral (LAT) view of X-ray images of the knee at pre-injection and post-injection (Hidden in the picture is the patient’s private information). **b** Kellgren–Lawrence analysis of the joint space between control (*n* = 10 individuals) and experiment (*n* = 10 individuals) groups. Analysis of Variance (ANOVA) with LSD Post Hoc test was used for statistical analysis. The PRP treatment (Pre-treatment: KL, 2.00; 95% confidence interval [CI], 1.50–2.50. Post-treatment: KL, 2.00; 95% CI, 1.50–2.50), HA control group (Pre-treatment: KL, 1.90; 95% CI, 1.50–2.30. Post-treatment: KL, 1.90; 95% CI, 1.50–2.30). **c** Quantitative T2-weighted MRI scan of the knee cartilage, red parts show soft tissue in the knee joint. **d** Quantitative analysis of the ROI in T2 value between control (*n* = 10 individuals) and experiment (*n* = 10 individuals) groups. The PRP treatment group (Pre-treatment: Quantitative MRI ROI, 122.610; 95% CI, 75.509–183.659. Post-treatment: Quantitative MRI ROI, 145.490; 95% CI, 92.289–210.238). The HA control group (Pre-treatment: Quantitative MRI ROI, 63.680; 95% CI, 52.701–76.570. Post-treatment: Quantitative MRI ROI, 80.030; 95% CI, 64.964–98.677). Analysis of Variance (ANOVA) with LSD Post Hoc test was used for statistical analysis. A representative result from nine independent experiments is shown. **e**–**h** Gait analysis of the control group (*n* = 10 individuals) and experimental group (*n* = 10 individuals). **e** The walking speed of the PRP treatment group (Pre-treatment: Speed, 0.917; 95% CI, 0.850–0.980. Post-treatment: Speed, 1.089; 95% CI, 1.001–1.182) and HA control group (Pre-treatment: Speed, 0.984; 95% CI, 0.846–1.098. Post-treatment: Speed, 1.015; 95% CI, 0.943–1.088). **f** The Moment max of HA control group (Pre-treatment: Moment max, 0.303; 95% CI, 0.212–0.404. Post-treatment: Moment max, 0.329; 95% CI, 0.235–0.436) a little bit higher than the PRP treatment group (Pre-treatment: Moment max, 0.312; 95% CI, 0.223–0.427. Post-treatment: Moment max, 0.333; 95% CI, 0.272–0.397). **g**, **h** The Gait analysis of the PRP treatment group and HA control group. **i** Pain analysis between control (*n* = 10 individuals) and experimental (*n* = 10 individuals) groups. PRP treatment group (Pre-treatment: VAS score, 30.60; 95% CI, 21.11–40.40. Post-treatment: VAS score, 12.95; 95% CI, 9.45–17.05) had a higher reduction of pain than the HA control group (Pre-treatment: VAS score, 28.50; 95% CI, 21.20–36.40. Post-treatment: VAS score, 13.90; 95% CI, 11.00–17.40). Analysis of Variance (ANOVA) with LSD Post Hoc test was used for statistical analysis. All data are reported as the mean ± SD

To confirm the effects of Sema3A enriched PRP in maintaining cartilage metabolism, mild OA patients were randomly selected to a PRP treatment group (KL1-3, *n* = 10) and a hyaluronic acid (HA) control group (KL1-3, *n* = 10) (Table 2, Extended Table 1). After the recruitment and multiple injections, there was no difference in X-ray analysis or KL scores between the PRP treatment and HA control group (Fig. S8A; Fig. 6a, b). However, the PRP treatment group tended to retain more soft tissue in the knee joint compared to that of the HA control group (Fig. 6c, d). To explore knee function and neuropathic pain, we used the dynamic gait index and the pain questionnaire (Visual Analogue Scale) accordingly. We found that the PRP treatment group ended up having a higher walking speed than the HA control group (Fig. 6e, Supplementary Movies 1–4). The HA control group tended to achieve a little bit higher moment max than the PRP treatment group, which may be associated with the lubricant properties of HA (Fig. 6f). Lastly, we analyzed the angle of joint movement and found no difference in either knee torque or hip torque between the PRP treatment group and the HA control group (Fig. 6g, h). Interestingly, the PRP treatment group had a higher reduction of pain than the HA control group when pre-treatment and post-treatment questionnaires were compared (Fig. 6i). Based on the significant chondroprotective effect of the PRP treatment group, and that PRP is rich in Sema3A, we speculate that Sema3A expressed by chondrocytes might play an important role in protecting cartilage and preventing nerve ingrowth in cartilage.

**Table 2:** Summary of OA patients treated with platelet-rich serum containing sema3A and hyaluronic acid

Group	Patient No.	Age	Gender	Pre-treat			Post-treat		
				KL	Pain scale	Quantitative MRI	KL	Pain scale	Quantitative MRI
Hyaluronate acid group	1	54	F	2	19	42.5	2	14	44.5
	2	57	F	2	12	50	2	9	66.6
	3	47	M	2	17	94.5	2	11	148.4
	4	63	F	2	14	65	2	11	80.6
	5	66	F	2	25	100.2	2	25	100
	6	64	F	1	35	46	1	10	57.8
	7	68	M	2	35	70.1	2	12	75.1
	8	34	M	1	39	52.1	1	19	64.7
	9	68	F	3	39	47.3	3	19	85.2
	10	57	M	3	50	69.1	3	9	77.4
Platelet-rich plasma group	11	68	M	2	16	30.8	2	8.5	40.7
	12	34	M	2	6	141.4	2	4	162
	13	68	F	2	18	207	1	14	296.6
	14	57	M	2	19	335	2	17	336
	15	54	F	2	17	83.6	2	11	125.4
	16	78	F	3	50	102	3	10	110.4
	17	32	F	1	45	18.5	1	20	24.2
	18	49	F	2	43	43	2	9	74.1
	19	59	M	3	54	48.2	3	25	54.3
	20	54	F	2	35	116.6	2	15	131.2

**Fig. 7:**
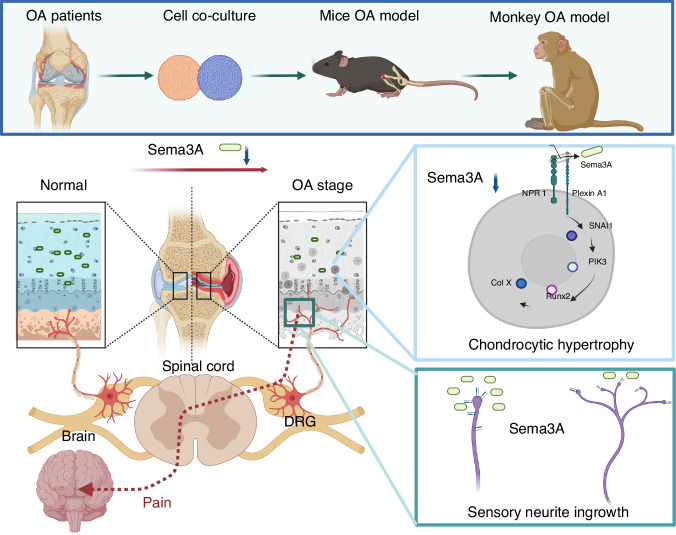
Neuronal guidance factor Sema3A plays a dual-functional role in the degeneration of knee cartilage. Schematic summarizing the mechanism of Sema3A exerting a dual-functional role in the knee degeneration process of mouse and Rhesus macaque OA models, and in patients with OA. Sema3A expression in chondrocytes regulates both nerve ingrowth and cartilage hypertrophy. (Created in BioRender)

Taken together, our data consistently demonstrated that Sema3A has a protective effect on cartilage degradation, validated by the organoid culture in vitro and Sema3A cKO mice, and in patients with OA by PRP in knee joint through intra-articular injection. We demonstrated that Sema3A maintains cartilage homeostasis via activating the PI3K pathway. The potential usage of Sema3A for OA treatment was validated in mouse, *Rhesus* macaque OA models. Our studies demonstrated that Sema3A exerts a critical role in inhibiting neurite ingrowth and preventing chondrocytic hypertrophy in OA cartilage, and could be potentially used for OA treatment (Fig. 7).

## Discussion

Knee joint usage become more frequent in the evolutionary transition from the knuckle-walking posture of apes to the erect posture of a modern human. The knee joint also became more erect and consequently transmits force in a more extended posture.^25–27^ Knee cartilage is the smooth surface on the bone ends in the knee joint. The cartilage from the tibia and femur become closer in proximity and are used more frequently in the erect than in the semi-erect and the crawling positions.^28,29^ Although this transition to being erect resulted in more economical locomotion, the proportion of cartilage to bone remained the same, and thus, cartilage is used more intensively during erect locomotion.^28,29^ Previous studies have shown the ingrowth of exogenous nerve factors in the remodeling of subchondral bone in an OA mouse model.^18^ Nevertheless, the mechanisms of neuronal infiltration into non-neural cartilage and the subsequent chondrocyte response remain elusive. The data presented in this translational study shows that Sema3A expressed in chondrocytes repelled synapse infiltration into cartilage and that Sema3A played a protective role in the maintenance of chondrocyte homeostasis. We found that chondrocytes in Sema3A^KD^ promoted neurite ingrowth and a hypertrophic phenotype, whereas chondrocytes in Sema3A^OX^ inhibited neurite ingrowth and maintained a chondrocytic phenotype. Interestingly, Sema3A conjugated with the Plexin-A receptor and regulated the PIPK3 pathway to inhibit the expression of collagen X, which is a marker of hypertrophy. Based on the previous reports and the observations in our previous study, the expression of Col X was mainly concentrated on the articular cartilage lesions and the cartilage around the tidemark.

We established an ACLT model to mimic OA pathology, in which there is a slow process of cartilage degeneration. The different manifestations of OA are obvious in this kind of model and met the requirements for the current study. The early nociceptive symptoms are partly associated with Sema3A induced-neural collapse and late-stage limitation of joint action is partially correlated with Sema3A-mediated-chondrocyte hypertrophy, which is one of the causes of osteophyte formation. This is interesting as it suggests dual roles for Sema3A in cartilage during different OA stages. Sema3A was first involved in the inhibition of neural infiltration and then supported chondrocyte homeostasis via the PI3K pathway. These both contributed toward the preventive effects of Sema3A and also the therapeutic potential, which was dependent on the injection time point during the pathological process. However, we found that chondrocytes become hypertrophic with the reduction of Sema3A in the late OA stages. This is partly associated with the anatomy of the synovial tissue that connects aneural tissue with neural tissue, which makes it more sensitive and vulnerable. Synovial tissue is thus worth investigating as a factor in the early initiation of cartilage degeneration in the future.

Our most important results were perhaps the therapeutic effects of Sema3A agents in the OA knee of both rodents and primates. In these species, different evolutionary processes are reflected in changes in the knee joint. For example, tibia and femur cartilage are used more frequently and are more sophisticated in fully-erect humans than in semi-erect monkeys or quadrupedal mice. We observed that treatment of mice knee joints led to the most obvious phenotypic changes: KL value, IF staining, and knee function all significantly improved post-operation. Treatment of *Rhesus* macaque knee joints was significantly different only in IF staining post-operation, and this difference was partly associated with OA severity. For OA patients, Sema3A containing PRP treatment only showed a tendency to increase the amount of soft tissues in the joint, and yet, pain relief was found following treatment. We speculate that these different treatment effects are partly associated with the evolution of the knee joint and that the tissue damage stemming from tibia and femur cartilage overuse in an upright knee posture makes this joint fragile, which leads to an intractable degeneration process that worsens with the age.

Interestingly, the Sema3A OS virus group showed a stronger tendency in the arrest of the OA process relative to the Sema3A protein group in OA *Rhesus* macaques. The efficient endogenous expression and longer half-life of Sema3A contributed to significantly increased Sema3A expression and an elevated level of neural collapse. Decreased expression of the neural factor Tau protein and Col X consistently gave weight to the premise that Sema3A gene therapy may be efficient in the prevention of nerve ingrowth and chondrocyte hypertrophy.

Studies with human OA patients are promising but have only occurred in small practice settings and need further validation through stringent drug testing. Because there is no available standard dose from the literature, for guidance we referred to the current common OA drug, HA. The clinical dosage of HA is mainly based on the volume of the articular cavity, which has not been standardized at present.^30,31^ HA treatment of OA lends only lubrication to the cartilage and lacks any specific therapeutic chondrocyte effect in the arrest of cartilage degeneration.^32,33^ Additionally, Sema3A has similar characteristics to anti-NGF, one of the novel monoclonal antibodies, which is still in the FDA approval process.^34^ There are several limitations in the current clinical study. First, PRP contains a variety of factors and we could not exclude the efficacy of other factors, if pure Sema3A agent was available commercially, its use as a therapeutic agent could be validated more efficiently. Secondly, due to the limitation of obtaining large numbers of patient recruitment, we would not be able to test the effects of Sema3A in both early- and late-stage OA in OA patients. Third, the follow-up (one month) may be short in this study and need to be optimized in a further trial. Further Sema3A studies may shed new light on the investigation of novel OA therapeutic approaches.^19,35^

This current study of Sema3A in chondrocytes contributes to our knowledge in three ways. Firstly, we reveal the mechanism of aneural homeostasis within cartilage in which the expression of Sema3A could fulfill its dual roles in the maintenance of normal chondrocytes via the PI3K pathway. This is an intracellular signal transduction pathway that promotes metabolism, proliferation, cell survival, growth, and angiogenesis in response to extracellular signals.^36,37^ Secondly, we demonstrate the clinical potential of Sema3A in the treatment of OA and this could be considered as an alternative treatment in the arrest of early cartilage denegation. This is consistent with the basic research outcomes, and the benefits are the efficacies of the VAS scores and radiography. There are no harms observed, including the infections post-injection. Finally, due to evolution, human knee joints are extended maximally in our upright posture, and overuse is inevitable. It is noteworthy that nociceptive symptoms are regarded as one of the early signals of nerve infiltration and cartilage degeneration, which is also thought to play a role in the attenuation the of degeneration process by the limitation of joint movement. This is consistent with the clinical syndromes for OA patients.

In summary, our results strongly suggest that neuron guidance factor Sema3A is involved in the maintenance of cartilage homeostasis and might offer a novel biological therapeutic option to arrest chondrocyte degeneration. Based on our observations of pain relief in OA patients, further improvements to our work should focus on an effective dosage of Sema3A to maximally affect pain relief and also consider the potential of human recombinant regimens. Last but not least, the timing of Sema3A treatment in different OA stages is important to consider, a factor that could facilitate safety and efficacy.

## Methods and materials

### Human participants

Human knee cartilage tissues were obtained from OA patients with knee arthroplasty (*n* = 10) and control trauma patients (*n* = 10, referred to as normal samples, Table 1). This was approved by the Ethical Review Board of West China Hospital Sichuan University (No. 2019149), and all patients gave written consent for the procedures undertaken and samples taken. The study of OA patients enrolled in the clinical treatment was approved by the Ethical Review Board of West China Hospital Sichuan University (2018-101) and the Chinese Clinical Trial Registry (ChiCTR1900025005).

### Mice strains and generation

The experimental procedures for animals were performed following approval from the Ethical Review Board of West China Hospital Sichuan University (No. 2018138A) and Shenzhen Institutes of Advanced Technology (SIAT-IACUC-201013-NS-YF-A1463), Chinese Academy of Sciences. Eight-week-old wild-type C57BL/6 mice were purchased from the Experimental Animal Center of Sichuan University and Sema3A conditional KO mice were generated using the Cre/loxP system. We purchased Semaphorin 3a-floxed mice (RBRC01106) and Sox9-CreER^T2^ knock-in mice (RBRC05522), which have a tamoxifen-inducible Cre (CreER^T2^) at the Sox9 locus from RIKEN BioResource Center (Japan). These two mice strains were kept in the same cage and mated to produce the next generation. The toes of male mice were cut for use in a Polymerase Chain Reaction (PCR) test and then Sox9-CreER^T2^ was identified during screening; in Semaphorin 3a^flox/flox^ mice (Sema3A^KO^). The primers used to genotype the mice are listed in Table 3. Sox9-CreER^T2^: Semaphorin 3a^flox/flox^ mice received intraperitoneal injection of Tamoxifen (100 mg/kg body weight, Sigma T-5648) to knockout the Sema3A gene. Tamoxifen was diluted with corn oil (Sigma C8267) to make a concentration of 20 mg/mL. A control group received intraperitoneal injections of corn oil (Sigma C8267) without Tamoxifen (0.05 mL/10 g). One treatment group (Sema3A^KO^ Pre-ACLT) received Tamoxifen intraperitoneally for seven consecutive days before ACLT knee operations. Another treatment group (Sema3A^KO^ Post-ACLT) received Tamoxifen intraperitoneally for seven consecutive days after a 30-day period following ACLT knee operations. The targeting efficacy of Sema3A was approximately 91% after Tamoxifen administration (Fig. S5F). All mice were maintained on a 12 h light/12 h dark cycle and under pathogen-free conditions with four or fewer mice per cage and had free access to food and water. All experimental mice were randomly assigned to control or treatment groups.

**Table 3:** Sema3A and Cre primer sequences

Primer name	P1 (Intron 5)	P2 (Intron 3)
*Sema3A*	ACAACGCTTGCCTCGGGAGGTAAA	ATGGTTCTGATAGGTGAGGCATGG
	P1 (exon 3F)	P2 (CW-Cre)
*Cre*	CCAGATGGACCCACCAGTATCAG	TCCGGTTATTCAACTTGCACCATGC

### Establishment of OA mouse model

Anterior cruciate ligament transection (ACLT) and sham surgeries were performed on male mice aged 8 weeks. ACLT was performed on the right knee and sham operations on the left knee as a control. We anesthetized mice intraperitoneally with 5% chloral hydrate (8 mL/kg body weight, KESHI). For sham operations, the left knee joint was exposed following a medial capsular incision, which cut the patellar tendon, and the surgical skin incision was closed with sutures. For ACLT surgeries, the anterior cruciate ligament was transected with microscissors after the opening of the right joint capsule. Tissue debris was then removed by saline irrigation before the skin incision was sutured.

### Hotplate sensitivity testing

Hotplate sensitivity testing was used to determine the response time of each mouse to react to a hotplate maintained at 54 °C. The latency period for a hindlimb response (for example, shaking, jumping, or licking) was recorded at several time points including before and after surgery. At least three measurements were taken at each time point for each mouse, with intervals of at least 10 min in between. The experimenter was blinded to the different mice groups.

### X-ray exposure and image analysis

X-ray images of osteoarthritis subjects (human and monkey) were obtained using a Digital Radiography device (SHIMADZU, 0.6/1.2P324DK-85; PlX102 X-ray source assembly) in the Department of Radiology, West China Hospital. Images were then evaluated using the Kellgren–Lawrence grading as follows: a score of 0 was given for no joint space narrowing or reactive changes; 1 for doubtful joint space narrowing and possible osteophytic lipping; 2 for definite osteophytes, possible joint space narrowing; 3 for moderate osteophytes, definite joint space narrowing, some sclerosis, possible bone-end deformity; and 4 for large osteophytes, marked joint space narrowing, severe sclerosis, and definite bone ends deformity. Images were independently evaluated by one radiologist and one orthopedic surgeon.

For mice, in vivo, 3D μCT (PerkinElmer, Quantum GX) was used pre-operation and post-operation at 8/12 weeks to quantify the cartilage changes associated with OA. For each animal group, at least four mice were imaged and analyzed. Mice were anesthetized using a Matrx anesthesia ventilator system (Matrx, VMR; Oxygen 1.0 L/min, isoflurane 150 mL/min) and then mice were immobilized bilaterally on hind limbs with adhesive tape. CosmoScan Database and Caliper Analyze (PerkinElmer, Analyzer 12.0; 90 kV, 8 μA, FOV: 25 mm, voxel size: 50 μm. Scan mode: high resolution, 14 min) were then used to reconstruct 3D images and analyze bone parameters. Images were analyzed by two investigators in a blinded manner.

### Histology

Articular knee-bone tissue from OA patients was fixed in 4% paraformaldehyde (Biosharp, BL539A) for one week at 4 °C. Twelve weeks following ACLT surgery, mice were sacrificed by intraperitoneal injection of excessive 5% chloral hydrate (8 mL/kg body weight, KESHI) and articular knee-bone tissues were separated and fixed in 4% paraformaldehyde for one week at 4 °C. Articular tissues from both humans and mice were decalcified with 10% EDTA-2Na (Amresco, 6381-92-6), pH 7.3, embedded in paraffin and then sectioned at 4 μm thickness.

We performed H&E staining (Sigma, 51275, and HT110216) and Safranin O Fast-Green Staining (Solarbio, G1371) for histological assessment. Histological OA severity of articular cartilage was evaluated using the Osteoarthritis Research Society International scoring system (OARSI score) as follows: a score of 0 for normal tissue; 0.5 for loss of Safranin O without structural changes; 1 for small fibrillations without loss of cartilage; 2 for vertical clefts down to the layer immediately below the superficial layer and some loss of surface lamina; 3 for vertical clefts or erosion to the calcified cartilage extending to <25%; 4 for vertical clefts or erosion to the calcified cartilage extending to 25%–50%; 5 for vertical clefts or erosion to the calcified cartilage extending to 50%–75%; and 6 for vertical clefts or erosion to the calcified cartilage extending >75%. Two investigators independently assessed OA severity.

### Immunofluorescence

Immunofluorescence (IF) staining was performed with the following primary antibodies: Semaphorin 3A (Abcam, ab23393; 1:200 dilution for humans and monkeys, 1:100 dilution for mice), Neuropilin 1(Abcam, ab81321; 1:250 dilution for humans, 1:100 dilution for mice), Plexin A1(Abcam, ab32960; 1:500 dilution for human, 1:200 dilution for mouse), Collagen X (Abcam, ab58632; 1:500 dilution for humans and monkeys, 1:600 dilution for mice), beta III Tubulin (Abcam, ab18207; 1:1 000 dilution for humans and mice), Tau (Millipore, MAB5430-C; 1:1 000 dilution for humans and monkeys, 1:500 dilution for mice), Anti-Phospho-Tau(T231)(Servicebio, GB114488-100, 1:500), F-actin (Abcam, ab205, 1:200 dilution for humans and monkeys, 1:200 dilution for mice) and GFP (Abcam, ab6556, 1:100 dilution for monkeys).

After dewaxing and hydration, the paraffin sections were incubated in a 3% hydrogen peroxide-methanol solution at room temperature without lighting for 10 min to inactivate endogenous peroxidase. Sections were washed (3 × 5 min in PBS), followed by blocking with 5% BSA Blocking Buffer (Solarbio, SW3015) for 20 min The primary antibody was incubated at 4 °C for 22 h. The next day, sections were rewarmed at 37 °C for 30 min, and then washed; (3 × 5 min in PBS). Donkey Anti-Mouse IgG (H + L) Highly Cross-Adsorbed Alexa Fluor Plus 594 (Thermo Fisher, A32744; 1:200 dilution) was used as a secondary antibody and incubated at 37 °C for one hour. Then, sections were washed (3 × 5 min in PBS), followed by DAPI counterstaining (Solarbio; C0065) for 5 min After washing (3 × 5 min in PBS), sections were sealed with Mounting Medium (Solarbio; S2100). Images were captured using a confocal microscope (Nikon, N-SIMS), and Image-Pro Plus 6.0 was used to count positive cells and calculate positive rates. For each group, at least two non-consecutive tissue sections were stained for each sample, and around 100 view fields were recorded in the quantification. Positive and negative controls in immunofluorescence staining (Fig. S9).

The quantitative calculation method for immunostaining is as follows: Select six to ten successfully stained slices for each tissue and take pictures. Select three different fields of view for each tissue slice and take pictures. For positive staining areas: we calculate the average proportion of positive areas in these three photos, which represents the positive rate of this slice. For positive cells: we calculate the average proportion of positive cells in the three photos, which represents the positive cells of this slice. After removing the area of non-tissue areas, measure the percentage of area with specific colors to the total tissue area using Image-Pro Plus6.0 (Media Cybernetics, USA).

### FISH staining

The FISH staining reagents and probes (M-Sema3a-647, M-SOX9-594) were purchased from the Spatial FISH company. Paraffin-embedded samples were prepared by cutting 7 μm thick sections, followed by dewaxing and gradient ethanol dehydration as per standard protocols. The sections were then treated with 5 μg/mL proteinase K for 5 min and fixed with 4% paraformaldehyde for 10 min, followed by three washes with PBS. Subsequently, the samples were sealed with 0.2% BSA and 2 mg/mL salmon sperm DNA for 30 min For the RNA in situ detection hybridization experiment, tissues were incubated in hybridization solution A containing the target probe pair at final concentrations of 30 nmol/L for 10–13 h at 40 °C. After incubation, the samples were washed gently with a series of gradient elution buffers to minimize noise and ensure positive signals. In the second step, the samples were incubated with hybridization solution B and the secondary probe for 2–3 h. The samples were then eluted with elution buffers A, B, and C as in Step 1. In the third step, the samples were incubated with hybridization solution B and the denatured tertiary probe for 2–3 h. After incubation, the samples were washed with elution buffers A, B, and C as in Step 1. In the fourth step, the samples were hybridized in solution C with signal probes for 1–3 h. After incubation, the samples were washed with elution buffers A, B, and C as in Step 1. Finally, the cells or tissues were stained with DAPI, dehydrated with gradient ethanol, sealed, and photographed to obtain FISH images.

### Von Frey testing

Absolute withdrawal thresholds were estimated using the ascending method with nylon monofilaments (Stoelting Touch Test). Animals were habituated in Plexiglas cubicles with a perforated metal floor for one hour prior to testing. Starting with the lowest von Frey filament (0.008 g), filaments were gently applied to the plantar surface of the hind paw until the withdrawal threshold was reached. A positive response was recorded when withdrawal, shaking, or licking of the hind paw was observed following filament application.

### Animal injection treatments

Before ACLT surgery, ten C57BL/6 male mice were injected intra-articularly with recombinant mouse Semaphorin 3A Fc chimera protein (R&D Systems, 5926-S3; 1:20 saline dilution, 50 μg/kg body weight) once per week for four weeks. Other groups were injected intra-articularly with lentivirus Rlv-CMV-sema3a-Efla-EGFP (*n* = 10), lentivirus Rlv-hv6-shRNA (Sema3a)-CMV-E6FP-T2A (*n* = 10), and anti-Sema3A antibody (Abcam, ab23393) (*n* = 10). At one month post-injection, knee micro-CT analysis and hotplate sensitivity test were performed. Consequently, mice were sacrificed for histology and IF testing.

*Rhesus* macaques (male, 17~24 y) were introduced and diagnosed with late-stage OA by X-ray. These animals received knee injection under anesthesia following approval from the Ethical Review Board of West China Hospital Sichuan University (No. 2018147A) and were divided into three groups: intra-articular injection of Hyaluronate acid (mean Mol. wt: 0.8~1.2 million, ARTZ^@^Dispo, JP), Sema3A protein (R&D SYSTEMS, 2150-S3) and lentivirus Rlv-CMV-sema3a-Efla-EGFP (BrainVTA, Wuhan, China) (*n* = 12, 4 monkeys in each group, 2 knees tested per monkey). No macaques were sacrificed during the experiment.

### OA patients treatments

From 2019 Sep to 2021 Feb, patients (aged 32~78 years) with primary OA (KL grade 1~3) were included from West China Hospital, Sichuan University. All the participants were divided randomly into a parallel-group study (allocation ratio = 1), including two groups: a hyaluronate acid group and a platelet-rich plasma group (including Sema3A protein) (Table 2). The full trial protocol can be assessed at http://www.chictr.org.cn/showproj.aspx?proj=41451. The sample size estimate was determined based on the primary outcome. Sample size calculations were based on the preliminary data with a minimally clinically important difference of 10% and a standard deviation of 15%. Considering α = 0.05 (two-sided), and power = 80%, the necessary sample size was calculated as *n* = 16. With a predicted dropout rate of 20%, the required sample size was calculated as *n* = 10 per group. Recruited patients were randomly allocated to two interventions (i.e., HA-only or PRP-only) based on a computer-generated randomization list, which was generated with the use of Randomization.com. One surgeon was responsible for all participants, and research personnel prepared the patient assignments, recorded practical details, and rechecked inclusion criteria. The randomization assignments were placed into sequentially numbered opaque sealed envelopes, which were inaccessible through the investigation period. An envelope was opened on the day of injection, and the appropriate study drug and control preparations were handled by search personnel not involved in patient care to ensure an identical appearance. The patients, trial participants, healthcare providers, outcome assessors, and data collectors were blinded to allocation and route of injection. 3 participants were stopped in each group due to the following reasons: (1) the patient is no longer suitable to continue participating in the trial; (2) lost to the follow-up; (3) There are some severe side effects or complications related to the trial. The date and reason for stopping the trial will be recorded with details. Eventually, the number of participants analyzed for the outcomes was 10 in each group.

Hyaluronate acid group patients (*n* = 10) were injected with 2.5 mL of hyaluronate acid agent (mean Mol. wt: 0.8~1.2 million, ARTZ^@^Dispo, JP) per week for 5 weeks following the standardized protocol. For the platelet-rich plasma (including Sema3A protein) group, patients received autologous peripheral platelet-rich plasma treatment (*n* = 10). Briefly, 45 mL of whole blood was drawn into a blood cell separator (WEGO, 20163661321) and mixed with 5 mL sodium citrate. The mixture was then centrifuged (1 200 r/min for 5 min) and red blood cells were discarded. A second centrifuge (1 200 r/min for 5 min) was then performed and 10 mL of PRP was collected from the lower layer. A sterilized needle was used to puncture 1 cm on the superolateral side of the upper margin of the knee joint. Successful needle entry into the joint cavity was confirmed when withdrawn without blood and then the 10 mL PRP was injected. Patients received four PRP treatments, once per week for 4 consecutive weeks. All the participants from the two groups received VAS score analyses (primary measure), X-ray, Quantitative T2-weighted MRI, and gait analysis (secondary measures) pre-injection and post-injection, respectively.

For the statistical methods used to compare groups for primary/secondary outcomes, statistical analyses were conducted using SPSS version 21.0 software. A P-value less than 0.05 was considered statistically significant. Primary and secondary outcomes were assessed with summary statistics, including measures of central tendency (mean ± s.d.) for quantitative data and numbers and percentages for qualitative data. Before breaking the randomization code, statistical analyses were conducted blinded.

### Gene-expression analysis

Total RNA from human chondrocytes was extracted using EasyPure RNA kits (Transgen, China), following the manufacturer’s instructions. The concentration and quality of RNA samples were determined using a B-500 biophotometer (METASH, China). Chondrocytes were cultured in complete DMEM/F12 containing 10% FBS and 1% Penicillin-Streptomycin. Neurons were cultured in complete Neurobasal (Gibco, USA) containing 2% B-27 (Gibco, USA), and 1% GlutaMAX (Gibco, USA). For quantitative RT–PCR, total RNA from human chondrocytes was reversely transcribed with ReverTra Ace qPCR RT Kit (TOYOBO, Japan), according to the manufacturer’s instructions. Gene-expression assays were performed with TransStart Green qPCR SuperMix (Transgen, China) and appropriate probes (BGI Tech, China). The murine housekeeping gene glyceraldehyde-phosphate dehydrogenase (GAPDH) was used as a control for data normalization. Fold changes were calculated using the 2^−ΔΔCt^ method. The expression of the target gene was tested with primers (Table 4).

**Table 4:** Primer names (marker genes) and sequences

Name	Forward	Reverse
*Col10a1(Homo)*	5′-ACCTTCTGCACTGCTCATCT-3′	5′-GTGTTGGGTAGTGGGCCTTT-3′
*PLXNA1(Homo)*	5′-GTGCAGTACACGTCCTGTGA-3′	5′-CCAGGACGCTCTCAGATTCC-3′
*PLXNB1(Homo)*	5′-GACACCATCTCCCAGGCAAA-3′	5′-CACAGTTGCTCCATCTGGGA-3′
*PTK2(Homo)*	5′-CAGTATTGGACCTGCGAGGG-3′	5′-TCCGCCCAATTCTTTTCTTCT-3′
*NRP1(Homo)*	5′-GGATGACAGCAAACGCAAGG-3′	5′-CAGTTGGCCTGGTCGTCAT-3′
*BCAR1(Homo)*	5′-TGGTAGTGTCAGGAGAGGGC-3′	5′-GATCTTGAGGCGGTTCCCAG-3′
*AKT1(Homo)*	5′-CGAAGACGGGAGCAGGC-3′	5′-CTCACGCGCTCCTCTCAG-3′
*PI3K(Homo)*	5′-TTGGAATAGTAGCAGGCGGC-3′	5′-GAAATGGTAGCTTCCCGAGGT-3′
*SNAI1(Homo)*	5′-AAGATGCACATCCGAAGCCA-3′	5′-CATTCGGGAGAAGGTCCGAG-3′
*Runx2(Homo)*	5′-GAATGCTTCATTCGCCTCACA-3′	5′-GTGCTGAAGAGGCTGTTTGAT-3′
*GAPDH(Homo)*	5′-ACAGCCTCAAGATCATCAGC-3′	5′-TTCAGCTCAGGGATGACCTT-3′
*Sema3A(Mus)*	5′-ACTCTCAGCTGCAGTAGTCAAT-3′	5′-ATTGTGCGGCCAGAGAAGTT-3′
*Esra(Mus)*	5′-TCTTGAACCAGCAGGGTGGC-3′	5′-GTTGAACACAGTGGGCTTGC-3′
*Adgrg3(Mus)*	5′-CAGTTTGGGACTGAGGGACC-3′	5′-GCCCACACTTGGTGAAACAC-3′
*Vegfa(Mus)*	5′-CGAGCTCATGGACGGGTGAG-3′	5′-GCCTGGGACCACTTGGCAT-3′
*GAPDH(Mus)*	5′-CAGGAGAGTGTTTCCTCGTCC-3′	5′-TTCCCATTCTCGGCCTTGAC-3′

The total RNA of each sample was extracted using TRIzol Reagent (Invitrogen)/RNeasy Mini Kit (Qiagen) total RNA of each sample was quantified and qualified using Agilent 2100 Bioanalyzer (Agilent Technologies, Palo Alto, CA, USA), NanoDrop (Thermo Fisher Scientific Inc.) and 1% agarose gel. One μg of total RNA with a RIN value above 7 was used for library preparation. Next-generation sequencing library preparations were constructed according to the manufacturer’s protocol (NEBNext® Ultra™ RNA Library Prep Kit for Illumina®). Then, libraries with different indices were multiplexed and loaded on an Illumina HiSeq instrument according to the manufacturer’s instructions (Illumina, San Diego, CA, USA). Sequencing was carried out using a 2 × 150 bp paired-end (PE) configuration; image analysis and base calling were conducted by the HiSeq Control Software (HCS) + OLB + GAPipeline-1.6 (Illumina) on the HiSeq instrument. The sequences were processed and analyzed by GENEWIZ. Firstly, reference genome sequences and gene model annotation files of relative species were downloaded from genome websites, such as UCSC, NCBI, and ENSEMBL. Secondly, Hisat2 (v2.0.1) was used to index a reference genome sequence. Finally, clean data were aligned to the reference genome via Hisat2 (v2.0.1) software. In the beginning, transcripts in Fasta format were converted from a known GFF annotation file and indexed properly. Then, with the file as a reference gene file, HTSeq (v0.6.1) estimated gene and isoform expression levels from the pair-end clean data. The DESeq2 Bioconductor package, a model based on the negative binomial distribution, was used for differential expression analysis. Estimates of dispersion and logarithmic fold changes incorporated data-driven prior distributions and Padj(P.adjust) of genes were settled. GOSeq (v1.34.1) was used to identify Gene Ontology (GO) terms that annotate a list of enriched genes with a significant padj less than 0.05. Also, topGO was used to plot DAG. We used in-house scripts to enrich the significant differential expression of genes in KEGG (Kyoto Encyclopedia of Genes and Genomes) pathways. KEGG is a collection of databases dealing with genomes, biological pathways, diseases, drugs, and chemical substances (http://en.wikipedia.org/wiki/KEGG).

### Cell harvest and spheroid culture in the Rocker system

Articular cartilage and subchondral bone were obtained from the medial and lateral condyles of the distal femur from patients diagnosed with knee OA who underwent total knee arthroplasty (Ethical license: 2019 (80)). The tissues were immediately stored in a sterilized specimen bag and merged into ice. Articular cartilage slices were aseptically dissected from subchondral bone and were immediately placed in a penicillin bottle filled with PBS (pH 7.2, Gibco). Cartilage was cut into small pieces with sterilized ophthalmic scissors. After centrifugation, the supernatant was discarded, and the cartilage debris was resuspended with PBS. This centrifuge-suspension process was performed three times. Chondrocytes were isolated by enzymatic digestion of cartilage debris using Pronase (0.4%, Merck) for one hour followed by digestion with Collagenase NB 4G (3.64 Pzu/mL, SERVA) for 4–6 h on a shaker (90 r/min). The digestion procedure was performed in a 37 °C, 5% CO_2,_ humidified cell culture incubator. Once digestion was complete, chondrocytes were separated with a nylon filter and resuspended with a culture medium (DMEM/F12 (1:1)) containing 5% fetal bovine serum (FBS, Gibco) and then seeded onto 25 T bottles (Corning). Cell culture was performed in an incubator at 37 °C under 5% CO_2_ with saturated humidity. The medium was changed three times per week. To generate a spheroid culture, harvested P1 human articular chondrocytes (HuACs) were resuspended in 10 mL DMEM: F12 (1:1) at a density of 5 × 10^5^ cells/mL and inoculated into a spheroid container (each 10 × 5 × 4 cm^3^) made of glass and surface-siliconized with Sigmacote (Sigma, UK). Under aseptic conditions, place newborn SD rats on their backs in ice D-PBS, cut the spinal canal along the dorsal midline of the spine, remove the exposed spinal cord, carefully extract the dorsal root segment with microscissors, and peel off the membrane and nerves. DRG neurons were cultured in complete Neurobasal (Gibco, USA) containing 2% B-27 (Gibco, USA), and 1% GlutaMAX (Gibco, USA), inoculated into a spheroid container (each 10 × 5 × 4 cm) made of glass and surface-siliconized with Sigmacote (Sigma, UK). The sealing strips on the container were cut into several pieces to allow for gas exchange. The spheroid containers were placed in the incubator and rocked continuously at 10 r/min using a Rocker system to induce spheroid formation. Representative 100 μL samples were removed from the spheroid container to determine spheroid diameter using a microscope (OBSERVER D1/AX10 cam HRC, ZEISS). The viability of HuAC spheroids was evaluated using the Fluoroquench fluorescence viability stain (One Lambda, Canoga Park, Calif., USA). 0.2 ng/mL and 1.2 ng/mL of Sema3A enriched plasma (50 μL) were added in 50 μL DRG culture medium and observed under a microscope 24 h later.

### Gait analysis, quantitative MRI, and pain inquiry questionnaire

Patients who had been enrolled in drug treatment underwent gait analysis pre- and post-treatment. In a gait laboratory, each patient walked at a self-selected pace on a 12-meter-long walkway. The patients wore 28 retroreflective markers to track the motion of the pelvis and each thigh, shin, and foot. An initial static trial was performed using 10 additional markers placed over the ankle, femoral epicondyles, and greater trochanter to determine segment orientations. Three-dimensional trajectories of the markers were collected at 290 Hz using a 10-camera motion analysis system (Oqus300, Qualisys; Gothenburg, Sweden). Ground reaction forces were measured at 540 Hz using two force plates (Bertec; Columbus, OH, USA) that were fitted directly underneath the walkway. Each patient performed five gait trials (the first two trials were for the purpose of adaption to the testing procedures and were not included in the final results) and was instructed to walk as naturally as possible looking straight ahead (Ewen et al. 2012). Inverse dynamic techniques and commercially available software (Visual 3D, C-Motion, Inc., Rockville, MD, USA) were used to calculate five gait variables: gait speed; single-limb stance time; sagittal (flexion/extension) plane knee ROM during walking and overall peak knee adduction moment (KAM) for both sides; hip joint (sagittal range of motion: max degree/mini degree), and knee joint (sagittal range of motion: max degree/mini degree). The characteristics and severity of knee pain in the OA patients were evaluated using two independent investigators before and after the intervention, which were followed by a manual chart. The level of the pain was quantified by a visual analog scale (VAS) and the characteristics were recorded and summarized into a final score, including site, nature, and persistence. For quantitative MRI, patients were turned to a supine position and scanned by a GE-SIGNA Creator-1.5 T. The scheme of the scanning includes 3-pl-T2*FGRE, T2-Mapping, and T2-MERGE.

### Statistical analysis and reproducibility

Statistical analyses were performed using IBM SPSS 22.0 and GraphPad Prism 10. The difference between the two groups was analyzed by t-test. One-way ANOVA or two-way ANOVA were used for multiple comparisons, followed an by LSD comparison test. A two-tailed Mann–Whitney U test and two-tailed Student’s t-test were also used for statistical analysis. *P-value* < 0.05 was considered statistically significant (*P < 0.05; **P < 0.01; ***P < 0.001). All data were expressed as the mean ± SD. All experiments have been successfully repeated with similar results at least three times.

## Supplementary information

Video1-HA pre

Video2-HA post

Video3-Sema3a pre

Video4-Sema3a post

Supplementary Figures

Extended table 1

## Data Availability

All data are available in the main text or the supplementary materials.
