# Supplementary material for: Neuronal guidance factor Sema3A inhibits neurite ingrowth and prevents chondrocyte hypertrophy in the degeneration of knee cartilage in mice, monkeys and humans
Source: Bone Res. 2025 Jan 2;13:4. doi: 10.1038/s41413-024-00382-0 (PMC11695747; doi:10.1038/s41413-024-00382-0)
Supplement: Supplementary file 5 — Supplementary Figures [file 41413_2024_382_MOESM5_ESM.docx]

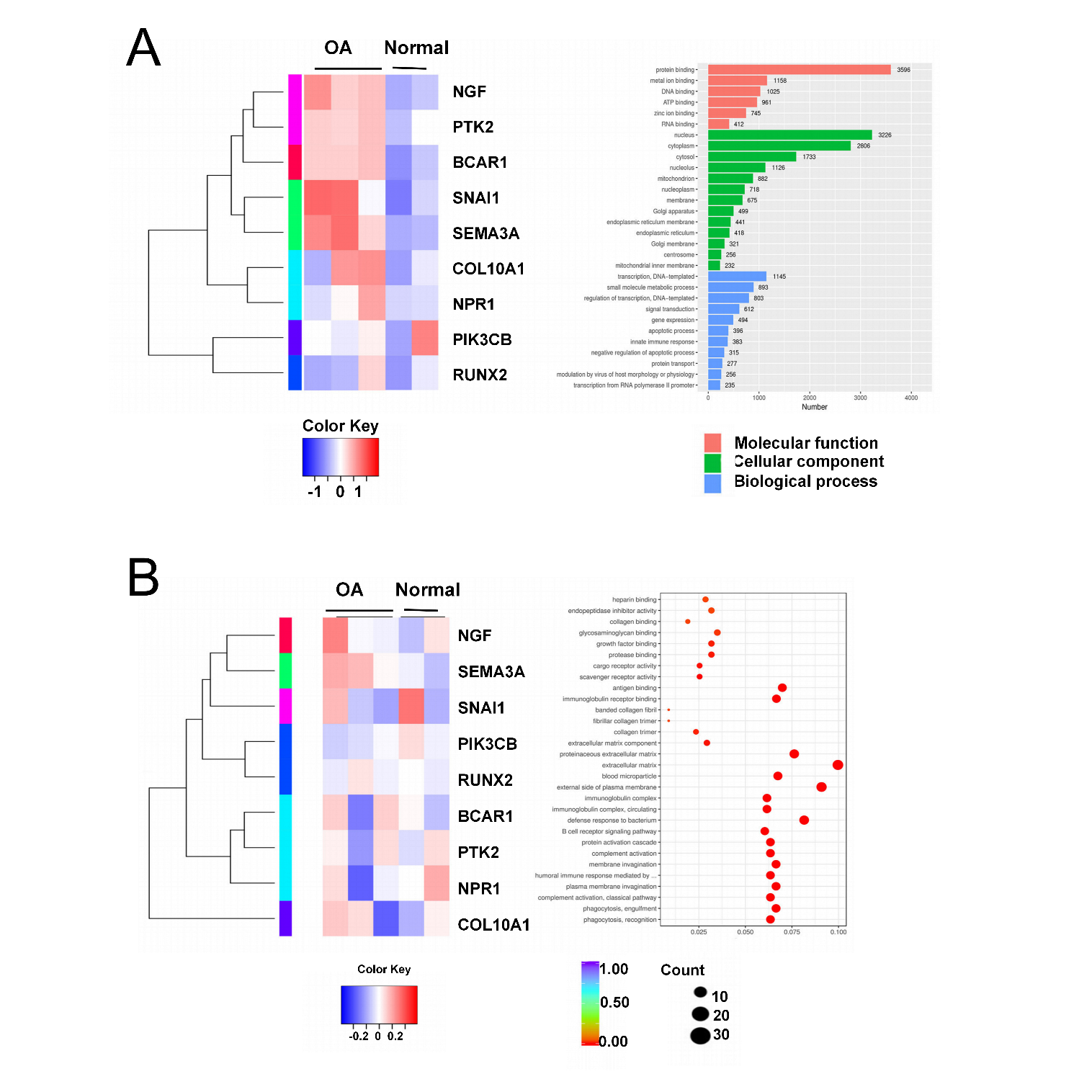


**Fig.S1**. Representative heat maps showing differential gene expressions between normal control (n=2 individuals, n=2 mice) and OA (n=3 individuals, n=3 mice) groups; **a,** human cartilage RNA-seq, **b,** mouse cartilage RNA-seq.


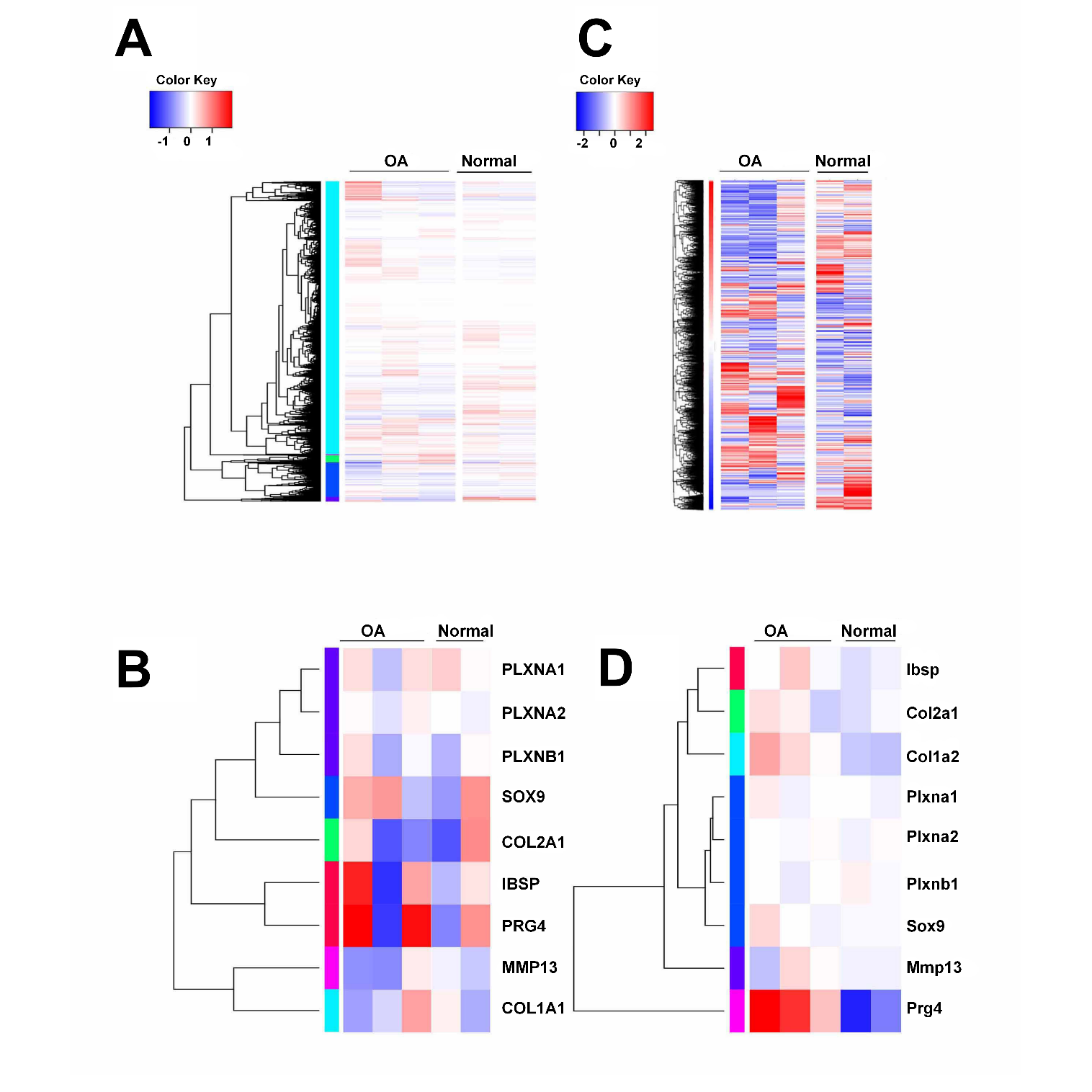


**Fig.S2.** Representative heat maps of RNA-seq in human and mouse cartilage, showing differential gene expressions between normal control (n=2 individuals, n=2 mice) and OA (n=3 individuals, n=3 mice) groups; **a, b** human cartilage RNA-seq, **c, d** mouse cartilage RNA-seq.


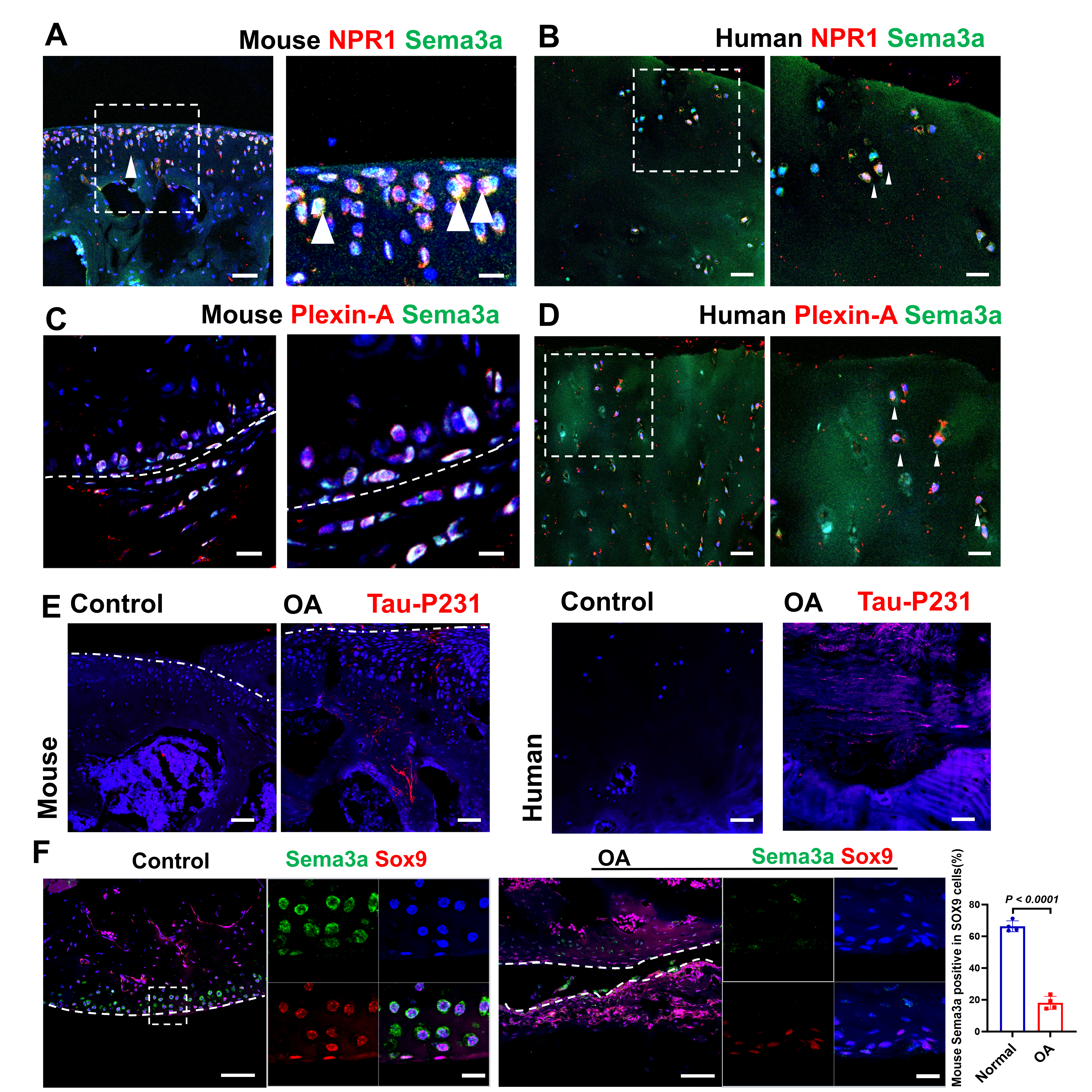


**Fig.S3.** Expression of NPR1, Plexin-A, Tau-P231 and Sema3A in the human and mouse articular cartilage.

**(a-b)**. NPR1 and Sema3A are co-expressed in mouse and human articular chondrocytes. (**c-d)**. Plexin-A and Sema3A are co-expressed in mouse and human articular chondrocytes. Scale bar, 50 µm. Scale bar in high magnifications, 25 µm. **(E).** Tau-P231 is expressed in mouse articular chondrocytes. **(F).** Sema3a is expressed in mouse articular chondrocytes through FISH staining. FISH staining of Semaphorin3A (Sema3A) and SOX9 in the normal control (n=4 mice), OA (n=4 mice) groups. Quantitative analysis of the number of positive Sema3a cells in cartilage was performed among groups. Scale bar, 50 µm.


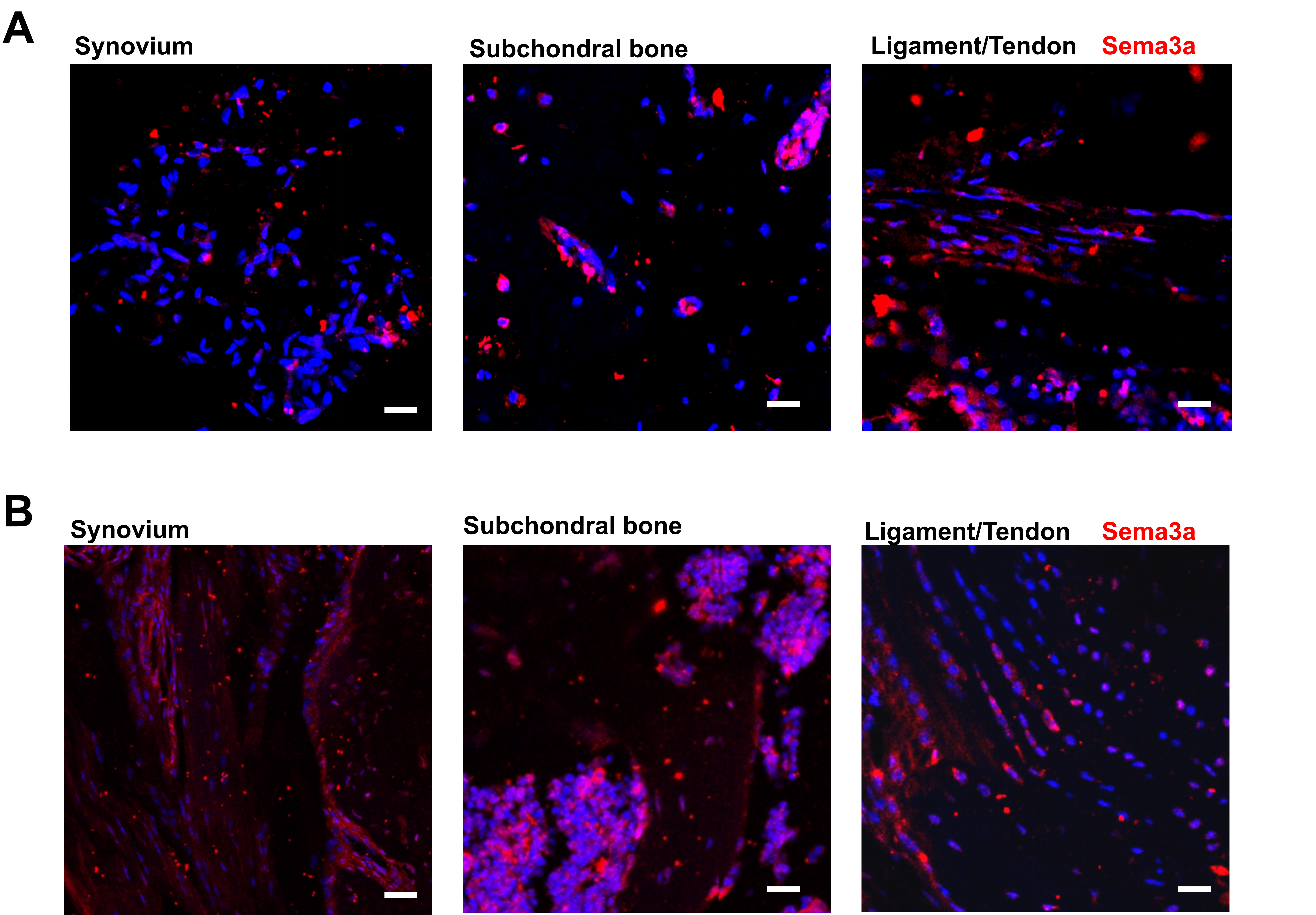


**Fig.S4.** Sema3a expression in various tissues of the joints.

**(a)**, The expression of Sema3A (red) in the synovium, subchondral bone and ligament/tendon of normal mouse joints.Scale bar, 25 µm. (**b)**. The expression of Sema3A (red) in the synovium, subchondral bone and ligament/tendon of OA mouse joints. Scale bar, 25 µm.


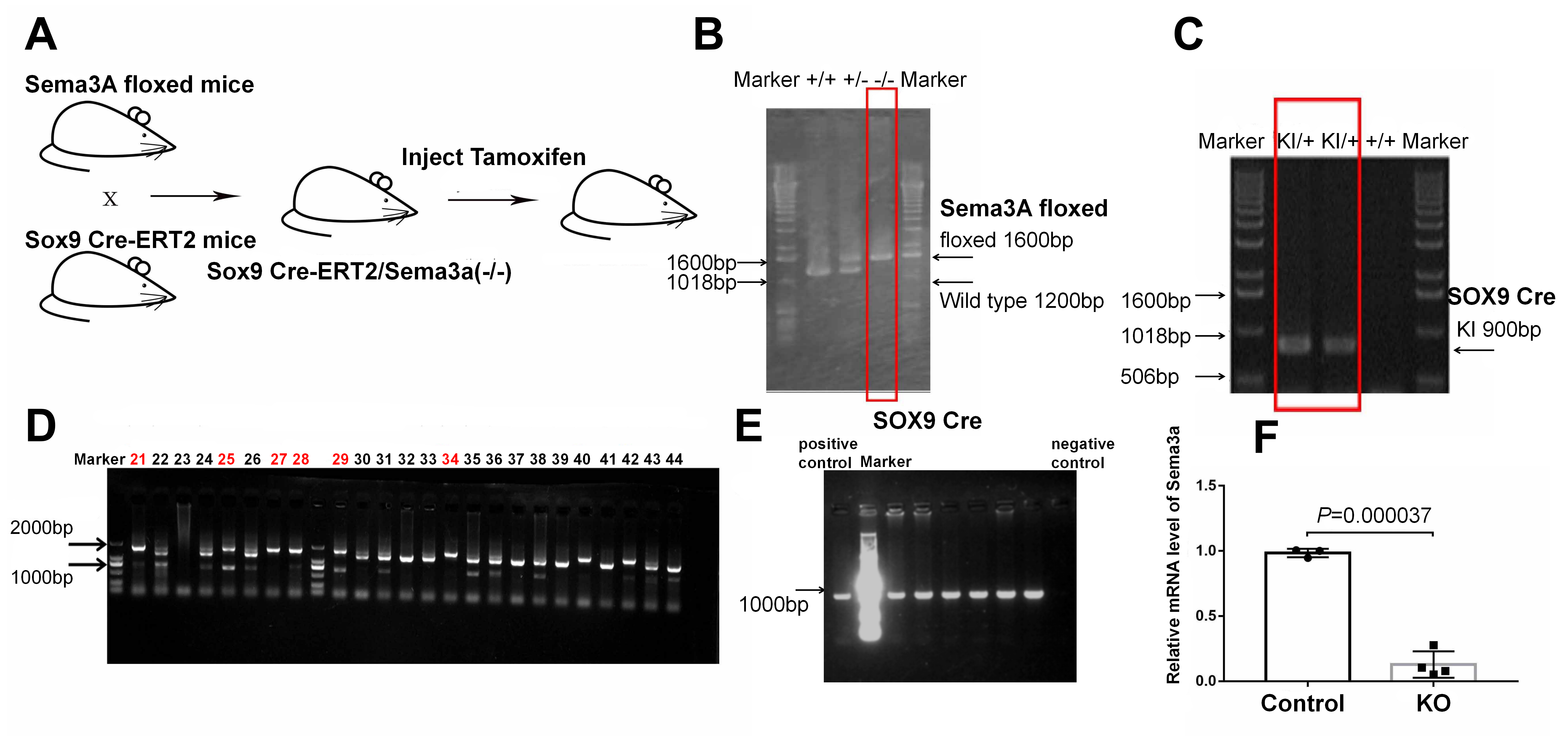


**Fig.S5.** Schematic program showing mice strains and generation.

**(a),** *Sox9-ER^T2^/Cre:Sema3a -/+* mice were generated by crossing heterozygote *Sox9-ER^T2^/Cre* strain with homozygote *Sema3a floxed* strain. *Sox9-ER^T2^/Cre:Sema3a -/-* mice were generated by crossing heterozygote *Sox9-ER^T2^/cre::Sema3a -/+* mice. (**b, c)** The genotype of the mice was determined by PCR analyses of genomic DNA isolated from mouse tails. The genotyping method followed the instructions for Sema3a floxed mouse (RIKEN: BRC 01106) and *Sox9-ER^T2^/Cre* mouse (RIKEN: RBRC 05522). (**d, e)** Mice numbered 21, 25, 27, 28, 29 and 34 were Sema3A-/-, and Sox9-Cre were positive. (**f)** Expression of Sema3A in chondrocytes was significantly suppressed.

**
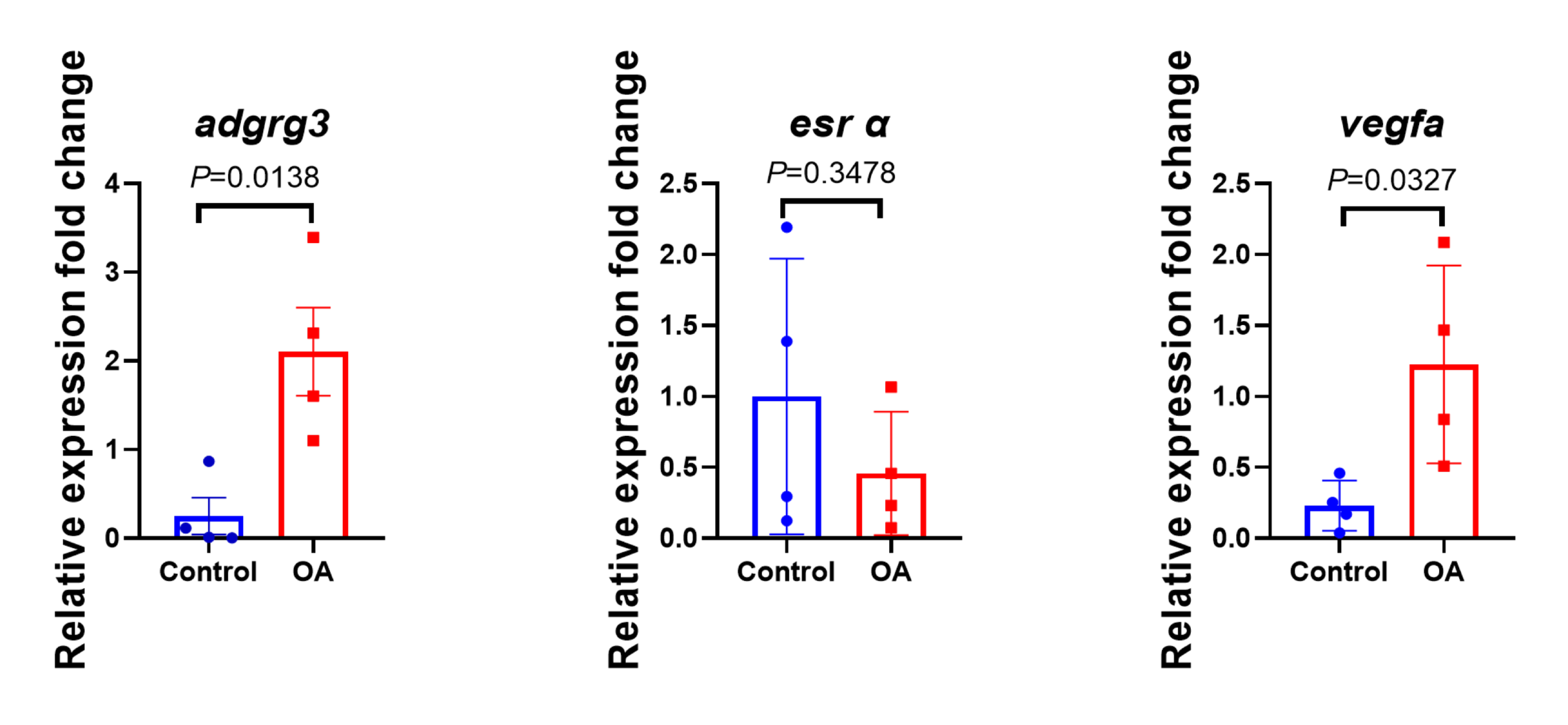
**

**Fig.S6.** The expression of upstream molecules in mouse chondrocytes. The expression of *adgrg3*, *esra*, *vegfa* were quantified by the real-time PCR in the control and OA mouse groups. (n = 4 for Ctrl, n = 4 for OA). A two-tailed t-test was used for statistical analyses. All data are reported as the mean ± SD.


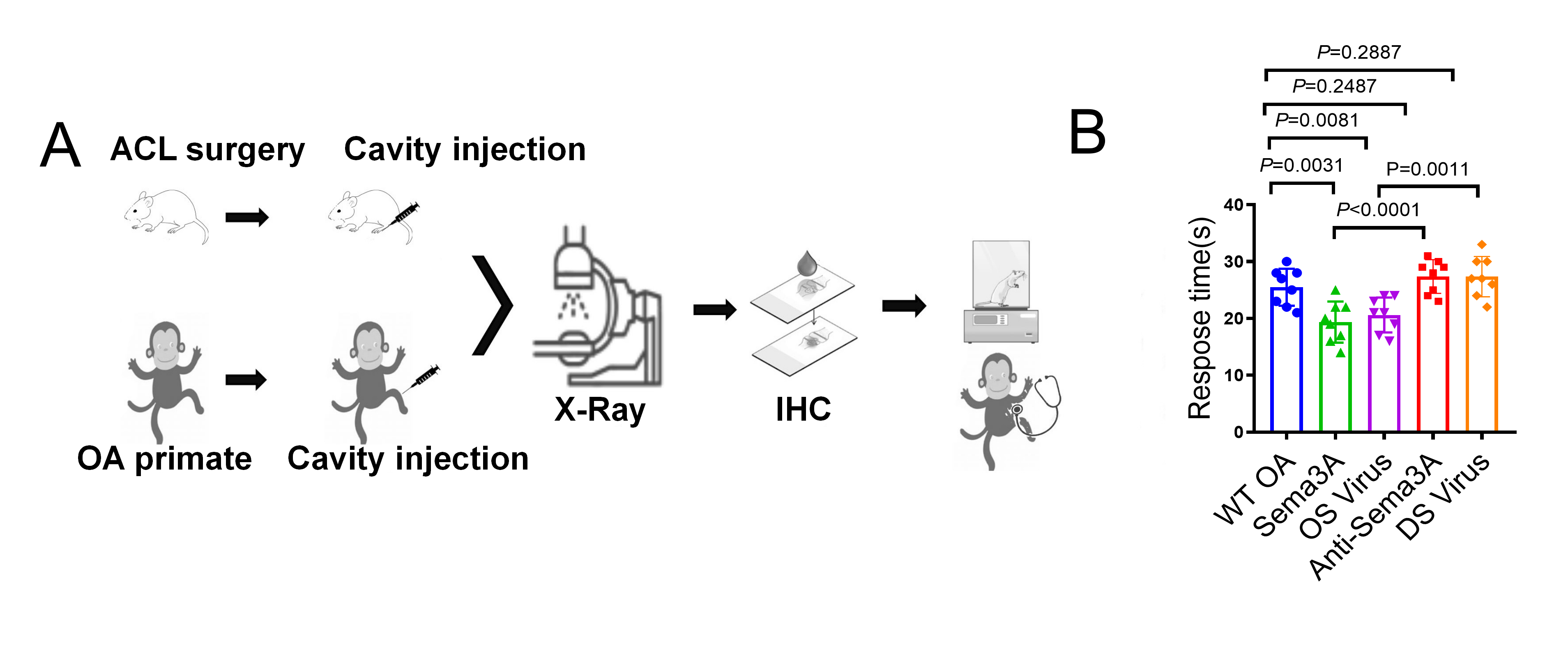


**Fig.S7.** Schematic showing that Sema3A treatment arrests OA progression in monkeys.

**(a)** X-ray, immunostaining and hot-plate test were used to evaluate the therapeutic potential of Sema3A in the treatment of OA in mouse and *Rhesus* macaque models. **(b)** Response time and pain scale in the hot-plate test among different groups of mice.


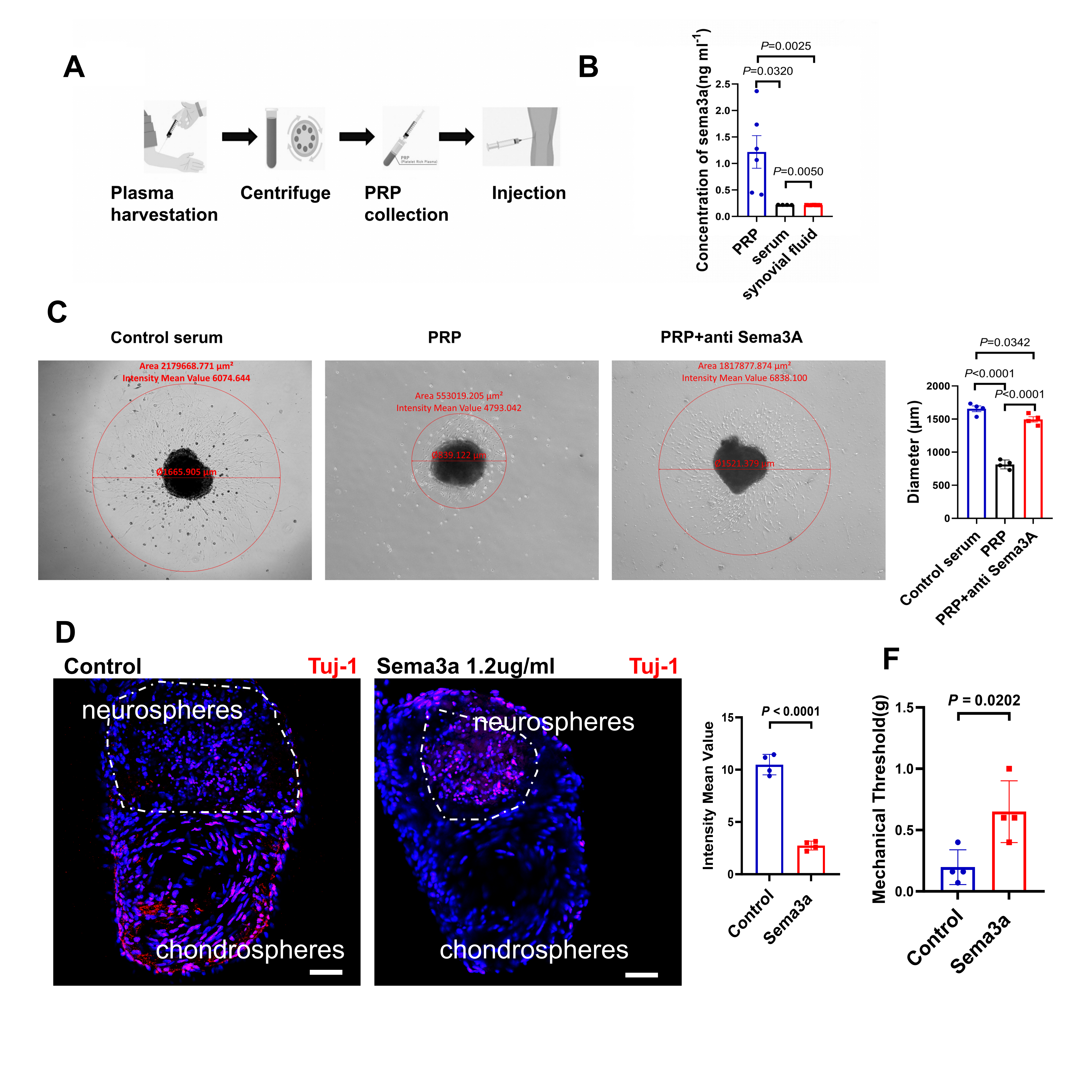


**Fig.S8.** Schematic showing that Sema3A treatment arrests OA progression in OA patients.

**(a)** Schematic showing the established autologous Sema3A refusion system. **(b)** Quantification of Sema3A in the control serum, sema3A enriched plasma (PRP) and synovial fluid. **(c)** PRP (containing 1.2 ng/ml of Sema3A ) significantly inhibited the ingrowth of the nerve fibers compared with the control serum，however 50 ng/ml anti-Sema3a anti-body significantly blocked the inhibitory effect of PRP(n = 4 for control, n = 4 for PRP, n = 4 for PRP+anti Sema3A ). **(D)** Sema3a(1.2 ng/ml) significantly inhibited the ingrowth of the nerve fibers compared with the control (n = 4 for control, n = 4 for Sema3A ). **(F)** Injected Sema3a (1.2ng/l) into the joint cavity of OA mice and assessed the pain response using Von Frey testing (n = 4 for control, n = 4 for Sema3A ).Two-tailed student's t-test test was used for statistical analysis. Mean with SD.


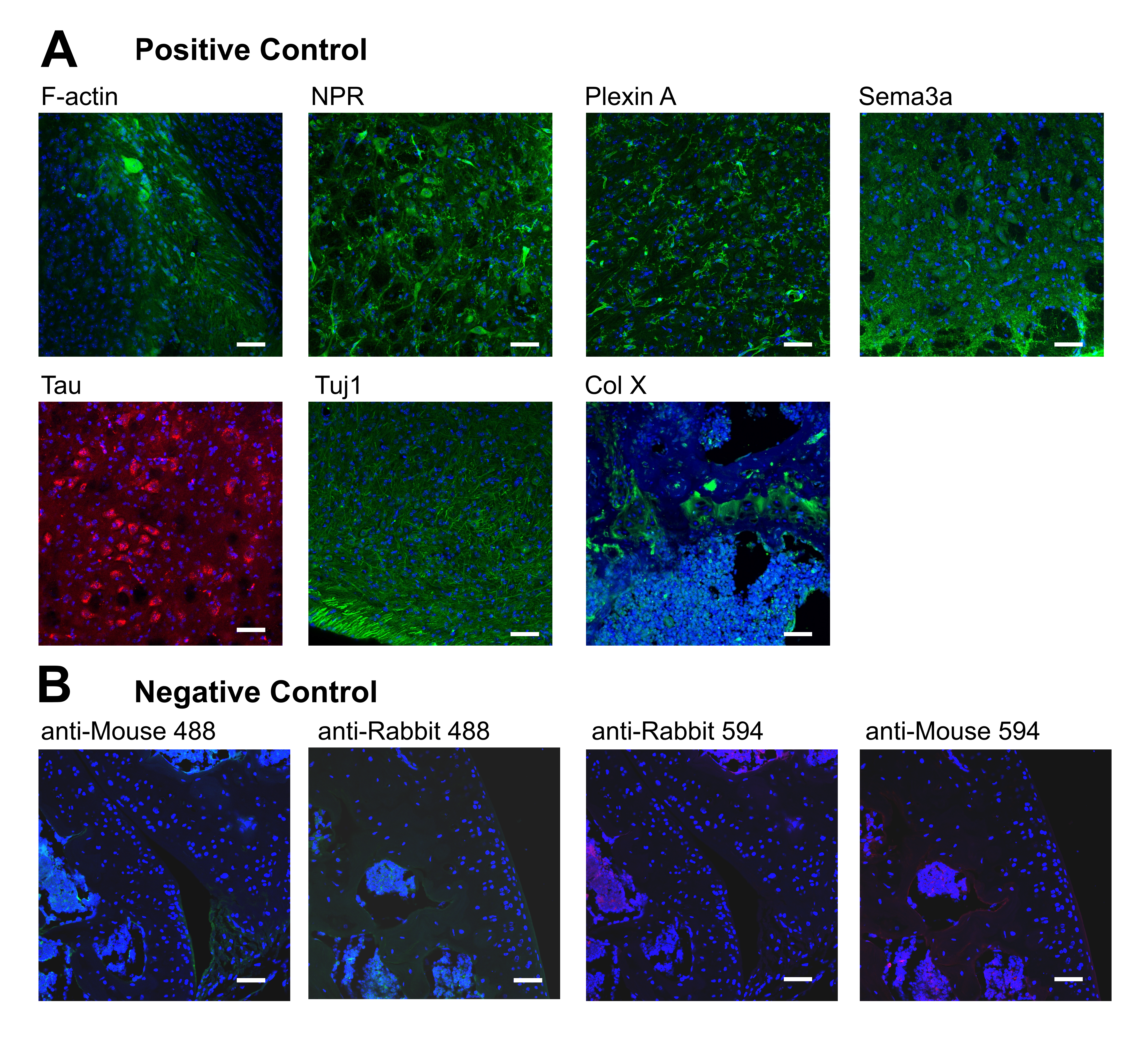


**Fig.S9.** Positive and negative controls in immunofluorescence staining.

**(a)** Positive control for F-actin, NPR1, Plexin A, Sema3A, Tau and Tuj1 primary antibody in brain tissue. Positive control for Col X primary antibody in bone tissue. **(b)** Negative control of secondary antibody in mouse joint tissue. Scale bar, 50 µm.

**Supplementary Movies**

Video1: Representative gait analysis of the HA group in pre-operation.

Video2: Representative gait analysis of the HA group in post-operation.

Video3: Representative gait analysis of the PRP group in pre-operation.

Video4: Representative gait analysis of the PRP group in post-operation.
