# Supplementary material for: Neuronal guidance factor Sema3A inhibits neurite ingrowth and prevents chondrocyte hypertrophy in the degeneration of knee cartilage in mice, monkeys and humans
Source: Bone Res. 2025 Jan 2;13:4. doi: 10.1038/s41413-024-00382-0 (PMC11695747; doi:10.1038/s41413-024-00382-0)
Supplement: Supplementary file 6 — Extended table 1 [file 41413_2024_382_MOESM6_ESM.docx]

**Extended table 1: Summary of OA patients in HA group and PRP group**

| Characteristics | HA Group (n=10) | PRP Group (n=10) | P value |
| --- | --- | --- | --- |
| Age (year) | 57.8±10.75 | 56±12.53 | 0.73 |
| Gender (Female/ Male) | 6/4 | 6/4 | / |
| Weight (Kg) | 57.6±8.58 | 64.15±9.20 | 0.11 |
| Kellegren-Lawrence score | 2.2±0.79 | 2±0.94 | 0.61 |
| VAS score | 28.5±12.84 | 30.6±16.54 | 0.75 |
| Red blood cell count(x10^12^/L) | 3.87±0.21 | 3.83±0.35 | 0.76 |
| Red blood cell count(x10^12^/L) in PRP | 0.41±0.20 | 0.55±0.30 | 0.24 |
| Platelet count (x10^9^/L) | 226.2±86.32 | 229.2±82.40 | 0.93 |
| Platelet count in PRP (x10^9^/L) | 528.2±118.30 | 578.8±135.86 | 0.39 |
